# Biocompatible fluorescent carbon dot-based nanoprobes for G-quadruplex targeting in cancer cells

**DOI:** 10.1039/d6ma00751a

**Published:** 2026-06-01

**Authors:** Teodoro García-Millán, Eliza Hunt, Nina M. Allen, Guilherme A. Marczak Giorio, Thomas A. A. Oliver, Javier Ramos-Soriano, M. Carmen Galan

**Affiliations:** a School of Chemistry, University of Bristol, Cantock's Close Bristol BS8 1TS UK tom.oliver@bristol.ac.uk fj.ramos@iiq.csic.es m.c.galan@bristol.ac.uk; b Glycosystems Laboratory, Instituto de Investigaciones Químicas (IIQ), CSIC – Universidad de Sevilla, Av. Américo Vespucio 49 Seville 41092 Spain

## Abstract

Carbon-based nanomaterials with intrinsic luminescence properties have emerged as promising tools in biological and biomedical research; however, tuning their photoluminescence properties for bespoke applications remains challenging. Herein, we report the design and synthesis of a new class of pH-responsive, red-emissive carbon dot-based nanoprobes (average core size 2–5 nm) functionalised with a naphthalene diimide (NDI)-based G-quadruplex (G4) ligand. These hybrid nanoprobes combine the favourable chemical and optical properties of CDs with the high selectivity of classical molecular ligands for G4 DNA. The resulting NDI-CDs exhibit excitation-dependent emission with distinct maxima at ∼450, ∼490, and ∼590 nm upon excitation at 350, 460, and 520 nm, respectively, in contrast to the excitation-independent emission of the parent CDs (*λ*_em_ ≈ 450 nm). Mechanistic investigations indicate that changes in the observed emission arise primarily from covalent surface conjugation with the NDI ligand that modifies the electronic structure of the CD surface and introduces additional emissive states. Control experiments rule out significant contributions from electron transfer or aggregation effects and instead support a mechanism involving surface-state modulation and pH-dependent protonation equilibria of the NDI moiety. Importantly, the NDI-CDs retain strong G4-binding affinity (*K*_d_ = 1.38 ± 0.25 µg mL^−1^) and simultaneously enhance ligand bioavailability and reduce cytotoxicity. Confocal microscopy studies of live cells treated with these nanoprobes show efficient cellular uptake, preferential localisation in mitochondria and nuclei, and significantly reduced cytotoxicity relative to the free ligand, confirming their suitability for biological applications, suggesting possible engagement with intracellular G4-associated structures under physiological conditions. Overall, this work establishes a versatile and biocompatible platform for the generation of carbon-based nanomaterials for multifunctional bioimaging and nucleic acid-targeting applications.

## Abstract 2

G-quadruplex ligand-functionalised carbon dots exhibit pH-responsive red emission, retained DNA-binding affinity and reduced cytotoxicity, offering a biocompatible platform for nucleic acid-targeted bioimaging.

## Graphical abstract

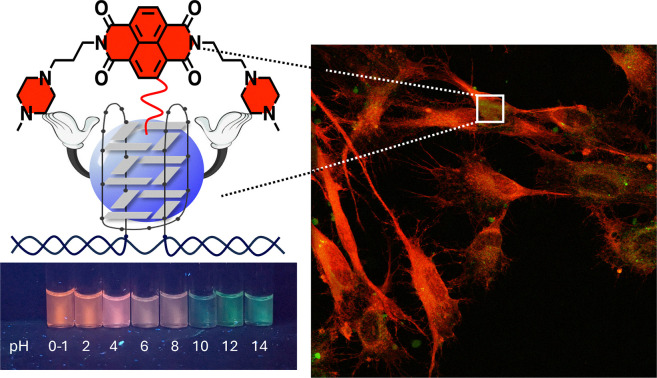

## Introduction

Fluorescence labelling of biomolecules is a common strategy to investigate their role and function in cells, tissues and organisms.^1^ The ongoing demand for improved photostability, sensitivity, and molecular specificity has driven the development of advanced fluorescent nanomaterials capable of operating under demanding biological conditions.^2,3^ Among these, carbon dots (CDs) have emerged as a promising class of carbon-based fluorescent nanomaterials. These materials offer excellent chemical and photochemical stability, straightforward and cost-effective syntheses, high water solubility, facile surface functionalization, and intrinsic biocompatibility, making them highly versatile as nanoscale platforms.^4^ Typically quasi-spherical and smaller than 10 nm, CDs share several desirable optical characteristics with the more toxic inorganic semiconductor quantum dots (QDs), including broad absorption spectra,^5^ composition-dependent tunable emission,^6,7^ and nanometre particle dimensions well suited to biological applications such as bioimaging. Beyond fundamental studies of their photophysics, CDs with different surface functionalizations have been explored in diverse scientific areas, including catalysis,^8^ LED^9^ or luminescence solar concentrator (LSC)^10^ devices, sensing,^11^ antimicrobial,^12^ antifungal^13^ and antiviral^14^ applications, as well as in gene delivery,^15^ cellular imaging,^16^*in vitro* theranostic platforms,^17,18^ and photosynthetic enhancement.^19^ Despite extensive research, the photoluminescence mechanisms of CDs remain poorly understood due to their complex structural and surface features, which hinder the rational design of materials with tailored optical properties.^24,25^ Additionally, most reported CDs rely on ultraviolet (UV) excitation, which can also induce substantial biological photodamage and strong autofluorescence interference.^20^

Recently, increasing attention has focused on the biological roles of G-quadruplex (G4) DNA, which is implicated in a wide range of cellular processes.^21–23^ This has driven substantial efforts to develop fluorescent tools for the detection and visualization of G4 DNA structures in living systems.^24^ G4s are non-canonical secondary structures formed by guanine-rich DNA sequences through Hoogsteen hydrogen bonding.^21,25^ These sequences are prevalent in telomeres, promoter regions, and untranslated sequences, where they play critical roles in gene regulation, genome stability, and replication, and are implicated in human diseases, including cancer.^21,26–28^ Previous strategies for G4 detection primarily relied on microscopy-based methods,^24,29,30^ such as immunofluorescence with G4-specific antibodies^31^ or small-molecule fluorescent probes,^32^ where the fluorescence properties are modulated upon G4 binding. However, antibody-based approaches require cell fixation and membrane permeabilization, precluding the study of G4 dynamics in living cells. In contrast, small-molecule fluorescent probes including thioflavin T,^33^ thiazole Orange,^34–36^ or *N*-mesoporphirin IX^37^ can cross cell membranes without permeabilization, thereby enabling visualization of G4s in their native environment. Nevertheless, many of these probes suffer from fluorescence quenching in aqueous media, and/or target selectivity, limiting their utility for live-cell imaging.^38^ Beyond small-molecule fluorogenic ligands, another important class of G4 probes relies on fluorescence modulation through chemical conjugation strategies. In this approach, a non-emissive G4 binder such as pyridostatin,^39^ or template-assembled synthetic G-quartets (TASQs),^40–42^ is covalently linked to fluorescent probes that do not directly interact with the nucleic acid. Despite their considerable cost, these probes have provided valuable insights into the subcellular localization of G4s and ligand interactions. Both covalent- (*e.g.* thiazole orange)^43^ and non-covalent- (*e.g.* hemin)^44^ modified blue-emitting CDs have been reported for the fluorescence detection of G4 DNA. However, their application has been largely restricted to *in vitro* systems, and their suitability for live-cell imaging has not been demonstrated. This limitation may arise from the intrinsic autofluorescence of biological samples, which can interfere with the detection of exogenous fluorophores emitting in the blue spectral region. Consequently, blue-emitting CDs may face sensitivity constraints in complex cellular environments, which reduces sensitivity and hampers their practical utility for intracellular G4 visualization.

To address this deficiency, we report the synthesis and characterization of a new class of pH-responsive, red-shifted emissive CDs functionalized with a G-quadruplex ligand for targeting of G4 structures in living cells. This approach combines the high selectivity of an established molecular ligand with the superior chemical and photophysical properties of CDs, resulting in an improved fluorescent platform. We demonstrate that ligand conjugation modulates the photophysical properties of the CD scaffold without compromising its binding affinity for G4 DNA, enabling effective targeting of G4 structures in live cancer cells under physiological conditions.

## Results and discussion

**Scheme 1:**
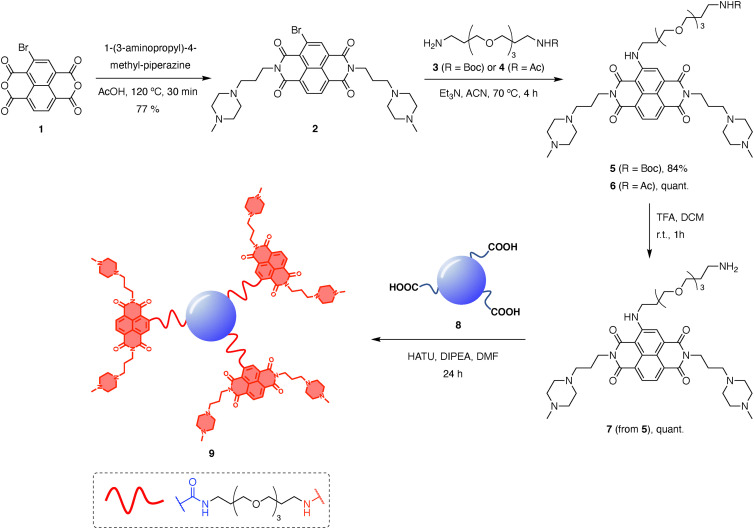
Synthesis of NDI-functionalised CDs 9.

To demonstrate proof-of-concept, trisubstituted naphthalene diimide (NDI) derivative 7, which features an amino triethyleneglycol spacer in its backbone as a chemical handle, was selected as the G4 targeting ligand. The compound was rationally designed based on a previously reported di-substituted methyl piperazine NDI scaffold developed by our group, which displayed high selectivity towards the human telomeric G4 in K^+^ buffer.^45^ Moreover, this scaffold exhibited higher cytotoxicity toward cervical cancer HeLa cells than the known anticancer drug doxorubicin, while being two-fold less toxic towards WI-38 (embryonic human lung fibroblasts) cells. The synthesis of trisubstituted NDI derivative 7 was achieved in three steps from 2-bromonaphthalene-1,4,5,8-tetracarboxylic dianhydride (1) as depicted in Scheme 1. Condensation with 1-(3-aminopropyl)-4-methylpiperazine under acidic conditions afforded the corresponding NDI 2 in 77% yield. Treatment of 2 with mono-Boc- 3^46^ or mono-acetate 4^47^ protected 4,7,10-trioxa-1,13-tridecanediamine (TTDDA) linkers under basic conditions afforded compounds 5 or 6 in 87% and quantitative yield, respectively. Finally, the tri-substituted NDI 5 was Boc-deprotected to give the desired amine-terminated NDI analogue 7 as the TFA salt in quantitative yield, ready to be conjugated to acid-coated CDs, whilst acetyl-capped NDI 6 was synthesized as a control for DNA biophysical studies. The molecular structure of all compounds was confirmed by NMR and mass spectrometry techniques (for full data, see Section S4.1.1). Concomitantly, acid functionalized CDs 8 were synthesized in a one-pot procedure from citric acid and ethylenediamine under microwave irradiation (domestic microwave oven, 300 W). The synthesis and full physicochemical characterization of these nanomaterials have been described in detail in our earlier works.^48,49^ Briefly, the resulting excitation independent blue-emissive CDs 8 have a high fluorescence quantum yield (QY) of 70% and transmission electron microscopy (TEM) revealed the nanoparticles were quasi-spherical with a graphitic core structure (lattice interspacing of 0.34 nm) and an average particle size between 2 and 5 nm.

NDI 7 bearing a primary amine group was then conjugated to carboxyl-functionalised CDs 8*via* a HATU-mediated amide coupling reaction in DMF in the presence of DIPEA. After 24 hours, the resultant NDI-functionalised CDs (NDI-CDs) 9 were treated with aqueous NaOH to remove any excess HATU bound to the CDs’ surface and Et_2_O to remove organic impurities, and neutralised with HCl. Purification by dialysis, followed by lyophilisation gave NDI-CDs 9. ^1^H, HSQC and Diffusion Ordered (DOSY) NMR spectroscopy analysis confirmed the successful conjugation of NDI moieties onto the CDs' surface (Fig. S1–S3). DOSY experiments revealed a radius of 1.8 ± 0.4 nm for NDI-CDs 9, which was two-fold larger than the radius of core CDs 8 (0.9 ± 0.1 nm) (Table S1), confirming their functionalization. Quantitative NMR was used to determine the percentage by weight (wt%) of NDI 7 within NDI-CDs 9, and it was found that 9 is composed of 55 wt% of 7 (see SI for more details).

**Fig. 1:**
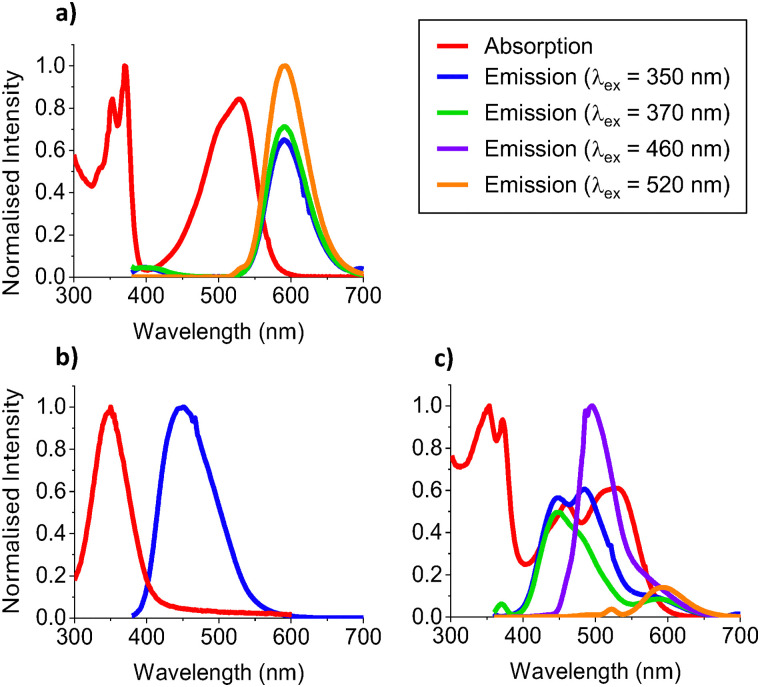
Overlaid absorption and emission spectra in H_2_O of (a) NDI (7) showing excitation independent emission for multiple excitation wavelengths, (b) CDs (8) exhibiting emission at 450 nm, and (c) NDI-CDs (9) showing maximum emission peaks at 450/490, 450, 490, and 590 nm, when excited at 350, 370, 460, and 520 nm, respectively, to evaluate whether certain wavelengths could excite exclusively the CDs or NDI.

Both NDI 7 and NDI-CDs 9 were fully characterised by steady-state UV-visible absorption and fluorescence spectroscopy. The absorption spectrum of NDI 7 shows a vibronic structure resulting from a π–π* transition at ∼350 and ∼370 nm and a peak at ∼520 nm associated with an intramolecular charge transfer state from the electron-donating amine substituent to the electron-deficient naphthyl ring (Fig. 1a). The latter might be attributed to hydrogen-bond interactions between the hydrogen atom of the secondary amine from the TTDA linker and the adjacent carbonyl oxygen in the NDI core.^50,51^ Excitation at each of these peak wavelengths gave rise to an emission band at ∼590 nm (Fig. 1a), as expected by Kasha's rule.^51^ Although some CDs exhibit excitation-dependent emission, this is not the case for unfunctionalized CDs 8 (Fig. 1b) derived from citric acid, *vide supra*, arising from electronic transitions associated with the CD core.^52^ Unlike the unfunctionalized CDs 8, NDI-CDs 9 exhibited an excitation-dependent emission spectrum, where the intensity of different emission bands varied strongly with the excitation wavelength (Fig. 1c). In addition, new bands were observed in the UV-visible absorption spectra at ∼460 nm and in the fluorescence emission spectra at ∼490 nm, which were absent in the spectrum of CDs 8 (Fig. 1b) or the NDI ligand 7 (Fig. 1a).

**Fig. 2:**
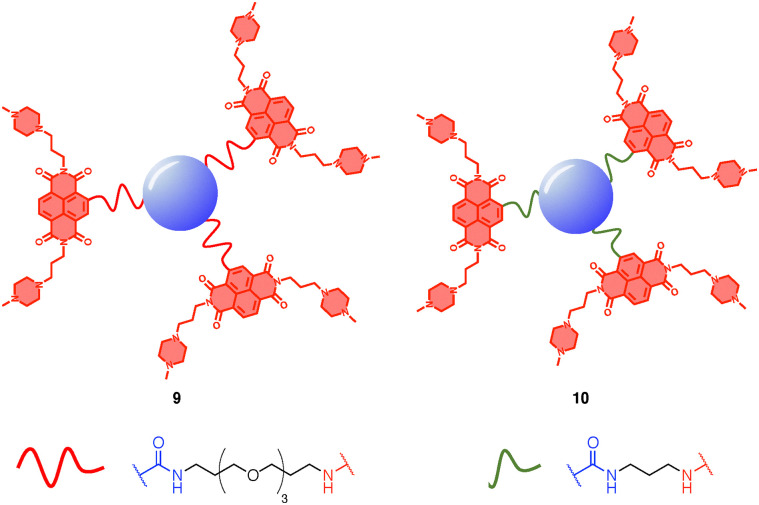
Structures of NDI-CDs with a long linker 9 (left) and short linker 10 (right).

Red-shifting the fluorescence of CDs is highly desirable for biological applications, as it can reduce interference from cellular autofluorescence and minimize photodamage compared to CDs that emit at shorter wavelengths.^53^ It has been proposed that this effect could be the result of electron/charge transfer,^54^ aggregation,^55^ or pH effects.^56,57^ To investigate whether the red-shifted emission of the NDI-CDs 9 was due to electron-transfer between the two moieties, a process that depends on the donor–acceptor distance, an analogous probe 10 (Fig. 2) with a shorter propyl linker between NDI and CDs core was prepared (for synthesis, see Scheme S1 and Section S4.1.1). Subtle variations were observed in the absorption spectra of the two probes (Fig. S4). Probe 10 (short linker) displayed a more intense absorption band at 460 nm than at 520 nm, whereas probe 9 (long linker) showed the opposite trend. However, their emission spectra were largely similar, indicating the excited state relaxation does not involve electron transfer (Fig. S4).

NDI aggregation-induced effects were next investigated since it has been reported that π–π stacking of the NDI cores can result in aggregation-caused quenching (ACQ).^55^ Thus, the emission spectrum of NDI 7 was measured at different concentrations ranging from 0.6 to 600 µM (to increase the likelihood of aggregation), resulting in only a small red-shifting of the *λ*_max_ from 577 nm to 598 nm (Fig. S5), which suggested that the new band observed in the emission spectra of NDI-CDs 9 was not related to aggregation effects.

**Fig. 3:**
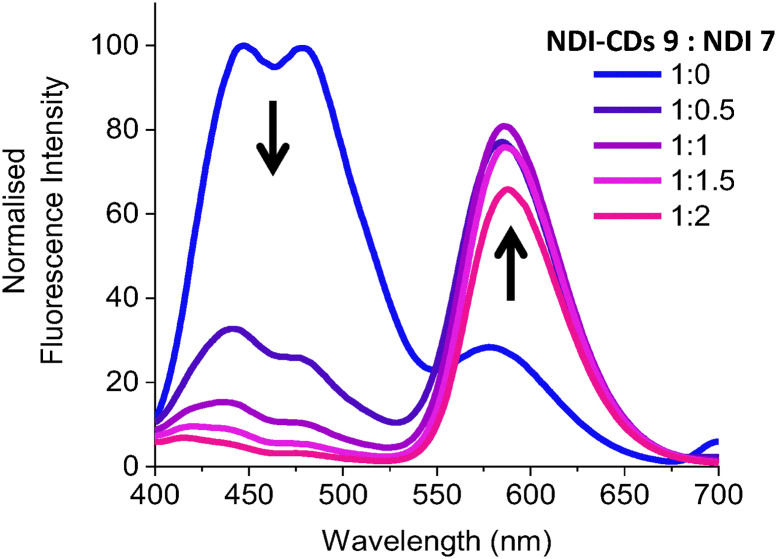
Fluorescence emission spectra (*λ*_ex_ = 350 nm, H_2_O) of NDI-CDs 9 at different equivalents of NDI 7.

To determine whether the fluorescence emission peak at 490 nm resulted from covalent amide conjugation of the NDI ligand 7 to the CDs as in 9, which might induce additional non-covalent interactions (*e.g.*, π–π stacking, ionic or H-bonding) between the NDI and the CD surface, or instead the shift could be attributed to completely non-covalent interactions between the two components, a fluorescence titration of NDI 7 to a solution of NDI-CDs 9 was carried out (Fig. 3). If the emission peak at 490 nm is due to non-covalent interactions between NDI 7 and the CD core, it was anticipated that the addition of increasing amounts of free NDI 7 would lead to an increase in the intensity of this peak. Conversely, if the emission at 490 nm is the result of covalent binding between NDI 7 and the CD core, as in NDI-CDs 9, it was expected that the addition of NDI 7 would not affect the 490 nm signal, but instead, an emission intensity increase at 590 nm would be seen. Aliquots of 7 were added to a solution of NDI-CDs 9, with the emission measured after each addition (Fig. 3). Addition of 0.5 equivalents (by weight) of NDI 7 resulted in a large decrease in fluorescence intensity at 450 and 490 nm, and an increase in intensity at 590 nm. Whilst saturation of the solution led to a slight decrease in emission intensity at 590 nm when introducing >1 equivalent of 7. This data indicated that the addition of exogenous NDI 7 quenches 9's fluorescence and that the observed new emission at 490 nm is thus likely the result of covalent conjugation of the NDI on the CD surface. These findings are in agreement with the previous results obtained by our group, in which it was demonstrated that surface functionalization *via* amide couplings significantly affects the physical and electronic structure of fluorescent CDs.^58^

**Fig. 4:**
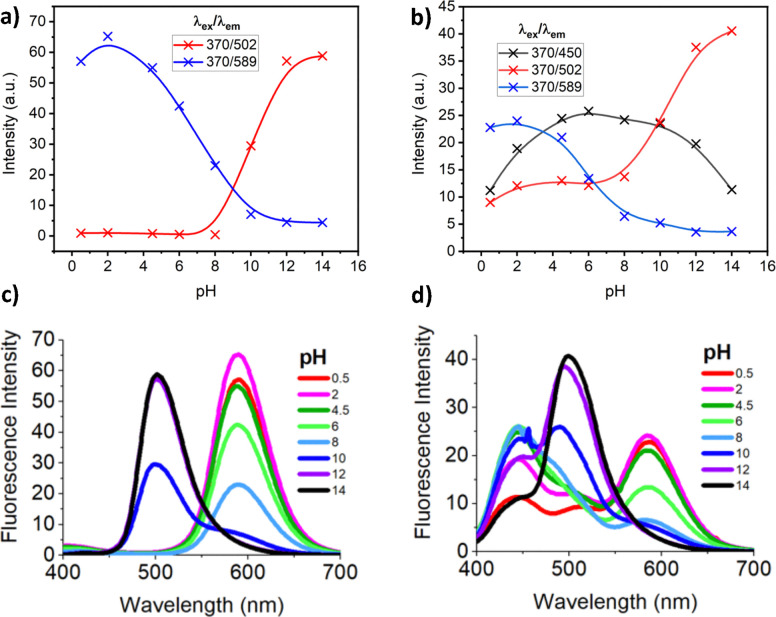
pH-dependent fluorescence intensity for (a) 7 at 502 and 589 nm (*λ*_ex_ = 370 nm); (b) 9 at 450, 502, and 589 nm (*λ*_ex_ = 370 nm). Fluorescence spectra of (c) 7 and (d) 9 at different pHs (*λ*_ex_ = 370 nm).

Considering previous reports of pH-sensitive photoluminescence in both CDs and core-substituted NDIs,^56,57^ we investigated whether the new emission band in NDI-CDs 9 at 490 nm was influenced by pH. Accordingly, solutions of NDI 7, CDs 8, and NDI-CDs 9 were prepared by mixing the different samples in aqueous solutions at different pHs ranging between 0.5 and 14. The absorption and emission spectra were measured for each compound at each pH at four different excitation wavelengths (*λ*_ex_ = 350, 370, 460 and 520 nm), see Fig. 4 and Fig. S6. Core CDs 8 showed pH-dependent emission, with the 450 nm peak (*λ*_ex_ = 350 nm) being quenched at both high and low pH, with a maximum emission intensity attained between pH 6 and 10 (Fig. S6). Both NDI 7 and NDI-CDs 9 displayed strongly pH-dependent fluorescence profiles, showing comparable spectral behaviour. Across all excitation wavelengths, the emission at 502 nm increased with pH > 8, whilst the opposite trend was observed for the band at 589 nm (*λ*_ex_ 520), which decreased as pH increased – with both emission wavelengths associated with the NDI component (Fig. 4a, b and Fig. S6). Interestingly, although the pH-dependent behaviour of 7 and 9 was similar, the presence of CDs in 9 contributed to an additional fluorescence band (*λ*_em_ 450 nm), which gives rise to a distinct pH-dependent colour fingerprint. Notably, the dominant emission wavelength (*λ*_max_) at low pH is 589 nm, whilst at high pH, *λ*_max_ shifts to 502 nm (both associated with NDI); and at neutral pH, the apparent *λ*_max_ is 450 nm (characteristic of the CD emission) (Fig. 4b). This tuneable emission behaviour furnishes these probes as potential pH-responsive biosensors.

With the structural and fluorescence properties of the NDI-CDs probe 9 determined, *in vitro* biophysical studies were undertaken using G4 and duplex DNA sequences to evaluate the probes' ability to interact selectively with G4-DNA. Taking into account that analogous piperazine-substituted NDI of 6 demonstrated strong G4 binding selectivity towards telomeric G4,^45^ we expected NDI-CDs 9 would behave in a similar manner. To confirm this hypothesis, we carried out binding studies using circular dichroism spectroscopy, UV-visible absorption spectroscopy, and fluorescence titrations. The telo23 G4 sequence was chosen as a representative oligomer found in the human genome. This sequence adopts a hybrid topology, which represents the most physiologically relevant G4 conformation under K^+^-rich conditions present inside cells.^59^

**Fig. 5:**
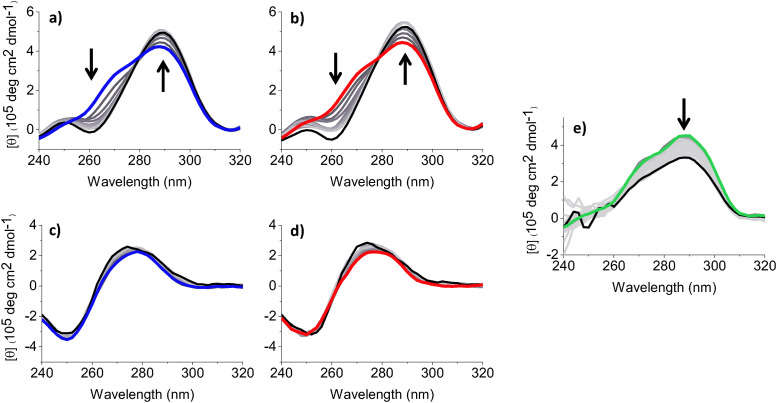
Circular dichroism titrations of a model hybrid telomeric G4 (telo23) with (a) 6 and (b) 9, duplex DNA (ds26) with (c) 6 and (d) 9, and (e) telo23 G-quadruplex with 8.

Circular dichroism spectroscopy was used to determine the effect of NDI-CDs 9 on the G4 topology, using free NDI 6 as a control (Fig. 5a and b). Ligand titrations were carried out with model human telomeric G4 (telo23) and a duplex DNA sequence (ds26) at pH 7.2 in a 100 mM KPhos buffer. Addition of both 6 and 9 to telo23 resulted in an increased circular dichroism signal at 290 nm and a reduction in the shoulder at 270 nm. Addition of larger amounts of 6 (10 equiv.) and 9 (7 equiv.) resulted in the formation of a negative band at 260 nm, which may suggest an induction of an antiparallel topology from the major hybrid G4 topology.^60^ This indicated that 6 and 9 perturbed the topology of G4 through strong binding interactions. Interestingly, addition of 6 and 9 to duplex DNA sequence, ds26, did not appear to cause any significant change to the circular dichroism spectrum (Fig. 5c and d), demonstrating a preference towards G4 DNA. As a control, circular dichroism titrations were carried out with core CDs 8 to telo23 (Fig. 5e). Interestingly, a decrease in the molar ellipticity was observed for CDs 8, suggesting a destabilizing interaction of the acid-coated CDs with the hybrid topology G4.

**Fig. 6:**
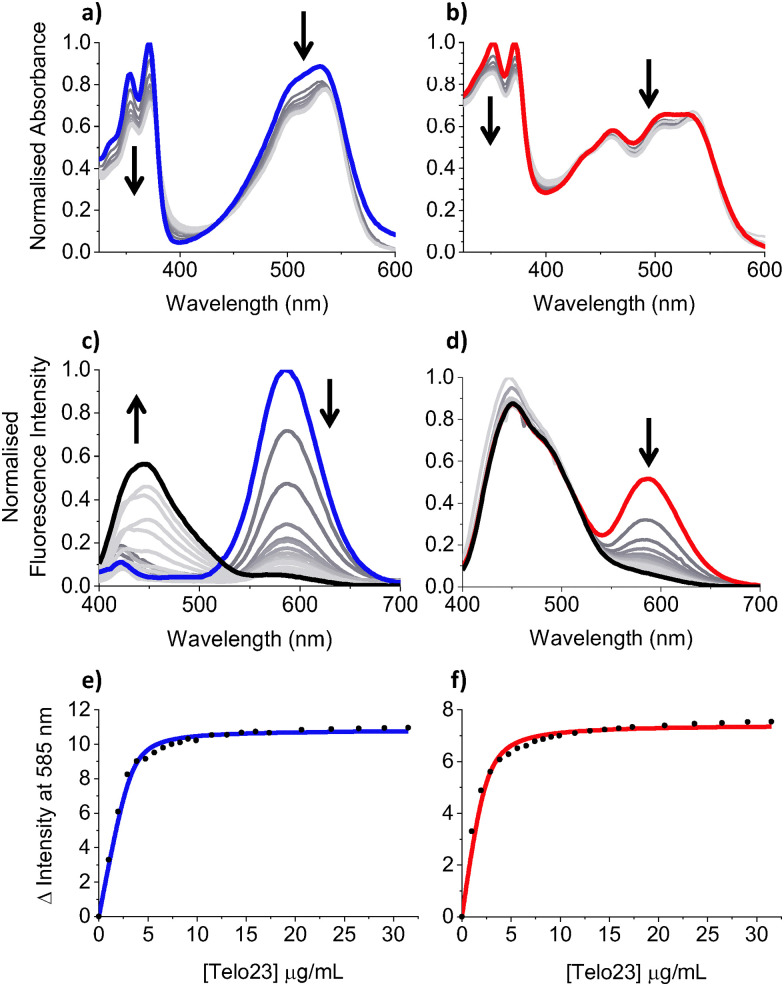
UV/visible spectroscopy titrations of (a) 6 and (b) 9 with telo23 and fluorescence titration (*λ*_ex_ = 370 nm) of telo23 with (c) 6 and (d) 9, alongside the corresponding binding curves (e) and (f), respectively.

To further confirm the binding observed for NDI-CDs 9 and NDI 6 to the human telomeric G4, UV-visible absorption spectroscopy titrations were measured. On addition of telo23 to 6, a decrease in absorbance was observed at 352 nm, 372 nm, and 532 nm, with the presence of isosbestic points at 372 nm and 340 nm (Fig. 6a). A similar result was observed with the addition of telo23 to 9, where an additional decrease in absorbance at 460 nm was detected (Fig. 6b).

Fluorescence titrations (at *λ*_ex_ = 370 nm) were conducted using similar conditions as those for the UV-visible absorption titrations. A decrease in fluorescence intensity at 585 nm was observed in the emission spectra of both NDI 6 and NDI-CDs 9 with increasing telo23 concentration, demonstrating the quenching of ligand fluorescence with DNA binding (Fig. 6c and d) likely due to electron transfer processes occurring between the NDI ligand and DNA. Interestingly, an increase in fluorescence intensity at 444 nm was observed in the spectrum of 6 as the concentration of DNA was increased, with a 10-fold increase observed at the maximum concentration of DNA (∼30 µM) (Fig. 6c).^61^ A slight increase in fluorescence intensity at 444 nm was also observed for NDI-CDs 9; however, the magnitude of enhancement is likely masked by the inherent broad emission band of the CDs at this wavelength (450–490 nm). The change in fluorescence intensity at 585 nm was plotted as a function of DNA concentration, and it was found that binding dissociation constants for NDI 6 and NDI-CDs 9 were *K*_d_ = 0.93 ± 0.17 and 1.38 ± 0.25 µg mL^−1^, respectively, with telo23 (Fig. 6e and f). Collectively, these results demonstrate that both NDI-CDs 9 and NDI 6 stabilize telo23 G4 and that the presence of a nanocarrier does not have a major impact on the interaction between the NDI ligand and the DNA.

**Fig. 7:**
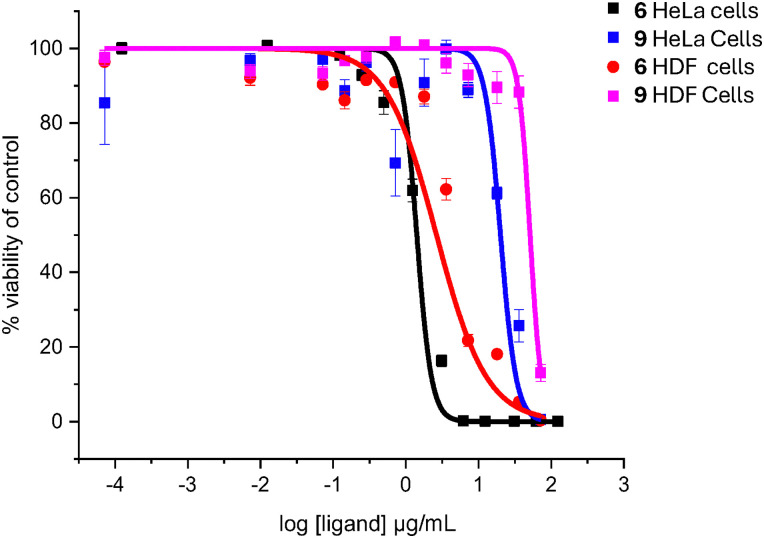
Dose–response curve fitted from alamarBlue toxicity assay with NDI 6 and NDI-CDs 9 on HeLa and HDF cells.

Ligand selectivity of NDI-CDs 9 towards G4s was carried out using a FRET melting assay with three cancer-associated G4-forming sequences and a parasitic sequence: an antiparallel 22-mer human telomeric G4 annealed in a sodium-rich (telo22-Na^+^), a hybrid 22-mer in potassium rich buffer (telo22-K^+^), a parallel 23-mer G4 from the c-Myc oncogene promoter region (c-Myc), a parallel parasitic sequence EBR1 and a duplex sequence (F10T) as control. The melting assay was carried out in a range of concentrations of 9 (1–10 µg mL^−1^) (see SI for details, Fig. S7). NDI-CDs 9 showed clear selectivity for the hybrid topology of telo22, with some moderate stabilization of the c-Myc sequence and no stabilization of the duplex sequence, consistent with previous observations for methyl piperazine NDI ligands (Fig. 7).^45^

**Fig. 8:**
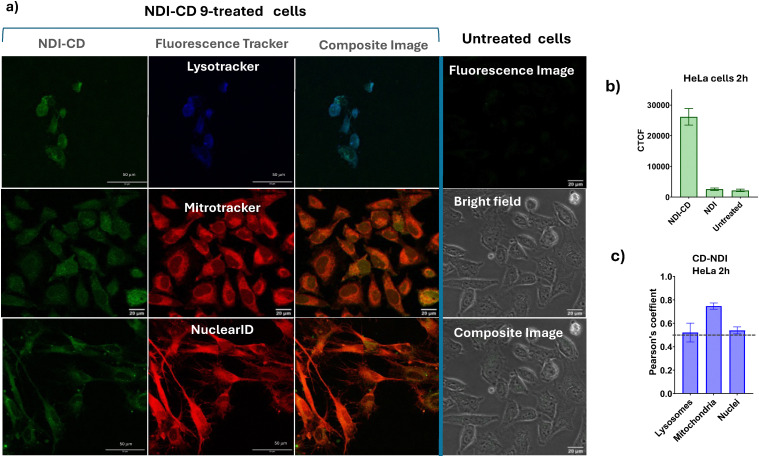
(a) Confocal fluorescence microscopy images of NDI-CDs 9 treated HeLa cells (250 mg mL^−1^) for 2 h, showing fluorescence channel for NDI-CDs 9 (green), fluorescent tracker (Lysotracker (blue), Mitotracker (red) or NuclearID (red) and composite images for each experiment. Images were compared to untreated cells and corrected for background autofluorescence. (b) Average corrected total cell fluorescence (CTCF) value obtained for NDI-CDs 9 (1), free NDI 6 (2) and untreated cells control (3) after 2 h incubation (*λ*_ex_ = 370 nm, *λ*_em_ = 589 nm). (c) Pearson's colocalization values for NDI-CDs 9 with Lysotracker, Mitotracker and Nucleus ID trackers after 2 h incubation and relative to untreated cells.

To determine the cellular uptake and cytotoxicity profile of the probes, cell viability in cervical cancer HeLa cells and healthy human dermal fibroblast (HDF) cells was assessed using the alamarBlue assay. For this assay, cells were incubated with free NDI 6, CDs 8, and NDI-CDs 9 (Fig. 8) in a range of different concentrations (1 × 10^−4^ µg mL^−1^ to 100 µg mL^−1^), and the cell viability was determined after 72 h, as previously described.^45^ It is important to note that dose–response curves were constructed based on NDI molarity, which was calculated for NDI-CDs 9 using quantitative ^1^H-NMR, which determined that 55 wt% of the sample corresponds to the NDI component. No toxicity was observed for HeLa or HDF cells after treatment with up to 100 µg mL^−1^ of CDs 8 (Fig. S8). Conjugation of NDI to the CDs, as in 9, resulted in a ∼15- to 20-fold decrease in cytotoxicity compared to NDI 6 alone in both cell lines (Table 1).

**Table 1:** EC_50_ (µg mL^−1^) values from alamarBlue toxicity assay with 6, 9 and 8 in HeLa and HDF cell lines after 72 h

	NDI 6	NDI-CDs 9	CDs 8
HeLa	1.41 ± 0.32	20.05 ± 2.59	>100 µg mL^−1^
HDF	2.64 ± 0.31	51.19 ± 16.89	>100 µg mL^−1^

The cellular uptake and intracellular localization of NDI-CDs 9 and free NDI 6 in live HeLa cells were investigated using confocal microscopy (*λ*_ex_ 460 nm (visible light) and *λ*_em_ 500 nm to ensure no overlap with fluorescent organelle tracker's emission profiles). Notably, the emission profile for 9 at 500 nm is influenced by intracellular pH changes, showing a reduction of intensity at low pHs (Fig. 4); the emission at 590 nm, which is enhanced at such pHs, is significantly diminished upon G4 binding (Fig. 6). For this reason, 500 nm was chosen as the best wavelength to monitor the targeting of G4 structures.

The optimal incubation time for probe internalization without affecting cell viability was determined by performing alamarBlue cell viability measurements at different time points (2–24 h) (Fig. S9). HeLa cells showed marked toxicity at 24 h (IC_50_ ≈ 10–20 µg mL^−1^), moderate toxicity at 15 h (50–80 µg mL^−1^), and minimal toxicity at 2 or 6 h. Given the requirement for high probe concentrations in live cell confocal imaging, a 2-hour time point was selected as the maximum incubation time for imaging studies, since confocal microscopy images after 6 h incubation with the probe showed significant levels of cell death.

Markedly, the average corrected total cell fluorescence (CTCF) value obtained for NDI-CDs 9 in HeLa cells was significantly higher than that of both free NDI 6 treated cells, at the same concentration as that present on 9 or untreated cells (Fig. 8b), suggesting that the CD component facilitates uptake. Internalization within cell organelles was determined by calculating the Pearson overlap coefficient (*R*)^62^ for each organelle marker and NDI-CDs 9 in HeLa cells that had been incubated with a 250 µg mL^−1^ solution of NDI-CDs 9 for 2 h before fixing with formaldehyde (Fig. 8a and c). NDI-CDs 9 were primarily localized in the mitochondria (*R* = 0.75), with moderate colocalization observed in the nuclei (*R* = 0.54) and lysosomes (*R* = 0.52). Since G4-DNA is predominantly localised in the nuclei and mitochondrial compartments,^31^ these results suggest that NDI-CDs 9 preferentially accumulate in these regions potentially *via* G4-DNA targeting before lysosomal clearance. We note that lysosomal uptake might be underestimated since this compartment is typically acidic and the emission of 9 at 500 nm is diminished at low pHs.

## Conclusions

We have developed a new class of pH-responsive, red-shifted emissive carbon-based nanoprobes functionalized with an NDI G4-ligand. The probes combine the excellent chemical and photophysical properties of CDs with the G4-targeting ability of the ligand and its inherent fluorescence properties. Molecular and structural characterization of the probes revealed that conjugation of a fluorescent functional motif such as NDI tunes the photophysical properties of the CDs without perturbing the ability of the ligand to target G4s and allows live cell imaging under more favourable visible light excitation wavelengths. Concomitantly, we enhance the ligand's bioavailability and mitigate its cell cytotoxicity, permitting its use at concentrations suitable for bioimaging applications. Overall, this work demonstrates proof-of-concept for a robust and adaptable design framework towards multifunctional nanoprobes for targeting G4 DNA in live cells, which could be adapted to other bioimaging applications.

## Author contributions

The manuscript was written through contributions of all authors. All authors have given approval to the final version of the manuscript. T. G. -M., E. H., G A. M. G., and N. M. A.: methodology, investigation, formal analysis, visualization, and data curation. T. A. A. O., J. R. S. and M. C. G.: conceptualization, funding acquisition, project administration, validation, supervision, writing – original draft, writing – review and editing.

## Conflicts of interest

There are no conflicts to declare.

## Supplementary Material

MA-007-D6MA00751A-s001

## Data Availability

All the underlying data to support the report findings are included within the manuscript and supplementary information (SI). The data include synthetic protocols, cytotoxicity and microscopy experimental details and characterization data for all compounds and outcomes. Supplementary information: spectral and other characterization data for all compounds and CD materials, full experimental details, materials, and methods, including structural characterization data and additional figures. Supplementary information is available. See DOI: https://doi.org/10.1039/d6ma00751a.
