# Supplementary material for: Biocompatible fluorescent carbon dot-based nanoprobes for G-quadruplex targeting in cancer cells
Source: Mater Adv. 2026 Jun 1;7(13):6809–17. doi: 10.1039/d6ma00751a (PMC13277541; doi:10.1039/d6ma00751a)
Supplement: MA-007-D6MA00751A-s001 [file MA-007-D6MA00751A-s001.pdf]

Supplementary Information (SI) for Materials Advances.
This journal is © The Royal Society of Chemistry 2026

Supporting Information

Biocompatible fluorescent carbon dot nanoprobes for G-quadruplex targeting in cancer cells

Teodoro García-Millán,[†a] Eliza Hunt,[†a] Nina M. Allen,[a] Guilherme A. Marczak Giorio,[a] Thomas A. A. Oliver,*[a] Javier Ramos-Soriano*[ab] and M. Carmen Galan*[a]

[a] *School of Chemistry, University of Bristol, Cantock's Close, Bristol, BS8 1TS, UK*

[b] *Glycosystems Laboratory, Instituto de Investigaciones Químicas (IIQ), CSIC – Universidad de Sevilla, Av. Américo Vespucio 49, Seville 41092 Spain*

[†] *These authors contributed equally.*

*Corresponding authors: tom.oliver@bristol.ac.uk; fj.ramos@iiq.csic.es and m.c.galan@bristol.ac.uk

1.	General …………………………………………………………………………	S1
2.	Synthesis and characterization………………………………………………	S2
3.	Biophysical studies……………………………………………………………	S14
3.1	Dichroism circular titrations………………………………………………	S14
3.2	UV/visible titrations ……………………………………………………………	S14
3.3	Fluorescence titrations………………………………………………………	S15
3.4	FRET melting assays……………………………………………………………	S15
4.	Biological studies………………………………………………………………	S17
4.1	Cell culture………………………………………………………………………	S17
4.2	Cytotoxicity………………………………………………………………………	S17
4.3	Confocal microscopy……………………………………………………………	S18
5.	References………………………………………………………………………	S19

1. General

Reagents and solvents were purchased as reagent grade and used without further purification. Reactions requiring anhydrous conditions were performed under N_2; glassware and needles were either flame dried immediately prior to use, or placed in an oven (150 °C) for at least 2 h and allowed to cool in a desiccator or under reduced pressure. CDs synthesis was conducted in either a domestic microwave (Wilko's Homebrand). For column chromatography, silica gel 60 (230-400 mesh, 0.040-0.063 mm) was purchased from E. Merck. Thin Layer Chromatography (TLC) was performed on aluminium sheets coated with silica gel 60 F_{254} purchased from E. Merck, visualization by UV light. NMR spectra were recorded on a Bruker AC 400 or AC500 with solvent peaks as reference. ^{1}H and ^{13}C NMR spectra were obtained for solutions in $CDCl_3$, MeOD and D_2O. All the assignments were confirmed by one- and two-dimensional NMR experiments (DEPT, COSY, HSQC and HMBC). Diffusion-Ordered (DOSY) NMR spectra was measured in D_2O at 500 MHz on a Varian spectroscope. Mass spectrometry was carried out by the University of Bristol Mass Spectrometry Service on Bruker microOTOF II or Thermo Scientific Orbitrap Elite instruments by electrospray positive ionisation (ESI+), or on a Bruker ultrafleXtreme 2 by MALDI ionisation. Infrared spectra were measured on a Perkin-Elmer Spectrum One FTIR machine with a scanning range of 4000 – 400 cm^{-1}. UV/visible spectra were recorded on an Agilent Cary 60 UV-Vis Spectrophotometer. Fluorescence measurements were conducted on a PerkinElmer LS45.

Abbreviations: CDs = carbon dots; G4 = G-quadruplex; NDI = naphthalene diimide; TTDDA = 4,7,10-trioxa-1,13-tridecanediamine; HATU: 1-[Bis(dimethylamino)methylene]-1*H*-1,2,3-triazolo[4,5-*b*]pyridinium 3-oxid hexafluorophosphate; DMF = *N,N*-Dimethylformamide; DIPEA = *N,N*-Diisopropylethylamine; ACN = acetonitrile; rt = room temperature; FAM = 6-carboxyfluorescein; TAMRA = 6-carboxy-tetramethylrhodamine; CTCF = corrected total cell fluorescence; DCM = dichloromethane; sat. aq. = saturated aqueous; TFA = trifluoroacetic acid; Boc = *tert*-butoxycarbonyl; quant. = quantitative; min = minute; h = hour.

2. Synthesis and characterization.

4-Bromo-2,7-bis(3-(4-methylpiperazin-1-yl)propyl)benzo[lmn][3,8]phenanthroline-1,3,6,8(2*H*,7*H*)-tetraone (2)

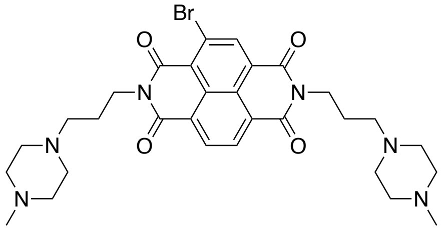

1-(3-Aminopropyl)-4-methylpiperazine (0.31 mL, 1.86 mmol) was added to a solution of 2-bromonaphthalene-1,4,5,8-tetracarboxylic dianhydride (**1**) (220 mg, 0.62 mmol) in acetic acid (7 mL). The reaction mixture was heated to 120 °C and stirred for 30 min. The solution was allowed to cool to rt and was then poured onto ice and neutralised with a solution of sat. aq. $NaHCO_3$. The crude product was extracted with DCM and purified by flash column chromatography on silica (20% MeOH in DCM) to yield yellow solid **2** (300 mg, 77%). ^{1}H NMR (500 MHz, $CDCl_3$) δ 8.92 (s, 1H), 8.80 (d, J = 7.5, 1H), 8.76 (d, J = 7.5, 1H), 4.32 – 4.21 (m, 4H), 2.50 (q, J = 7.0, 4H), 2.75 – 2.21 (br. m, 16H), 2.17 (app. d, 6H), 2.01 – 1.89 (m, 4H); ^{13}C NMR (126 MHz, $CDCl_3$) δ 162.7, 162.1, 161.9, 161.2, 138.5, 131.8, 131.0, 130.8, 128.8, 127.0, 126.2, 126.1, 125.9, 124.1, 56.2, 56.1, 55.2 (8C), 46.2 (2C), 40.1, 39.8, 25.0, 24.9; HR-MS (ESI-positive ion) *m/z* for $[C_{30}H_{37}BrN_6O_4]^+$ $[M+H]^+$ calcd: 625.2133, found: 625.2109. The characteristic bromide isotope pattern can be observed in the HR-MS; IR ν_{max} (cm^{-1}) (compressed solid) 2933 (w), 2792 (w), 1705 (m), 1662 (s).

Compound 5.

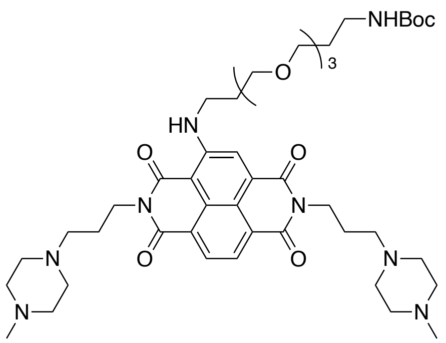

A solution of *tert*-butyl (3-(2-(2-(3-aminopropoxy)ethoxy)ethoxy)propyl)carbamate (**3**)[1] (0.30 g, 0.94 mmol) in Et_3N (1.5 mL) was added to a suspension of bromo-NDI **2** (0.20 g, 0.31 mmol) in ACN (3 mL) under a nitrogen atmosphere. The reaction mixture was heated to 70 °C and stirred

for 4 h. The solution was then cooled to rt, and solvent was removed under reduced pressure. The crude product was redissolved in DCM and washed with H_2O. The organic extracts were dried with $MgSO_4$, and the solvent was removed under reduced pressure. The crude product was purified by flash column chromatography on silica (20% MeOH in DCM) to yield trisubstituted NDI **5** (0.23 g, 84%) as a solid. ^{1}H NMR (500 MHz, $CDCl_3$) δ 10.06 (t, J = 5.5, 1H), 8.49 (d, J = 8.0, 1H), 8.19 (d, J = 8.0, 1H), 8.07 (s, 1H), 5.13 – 5.00 (m, 1H), 4.15 (t, J = 8.0, 4H), 3.7 – 3.57 (m, 14 H), 3.54 (dd, J = 6.0, J = 3.5, 2H), 3.48 (t, J = 6.0, 2H), 3.16 (q, J = 6.5, 2H), 2.61 – 2.21 (br. m, 16H), 2.17 (s, 3H), 2.14 (s, 3H), 2.04 (p, J = 6.5, 2H), 1.86 (p, J = 7.0, 4H), 1.70 (p, J = 6.5, 2H), 1.36 (s, 9H); ^{13}C NMR (126 MHz, $CDCl_3$) δ 163.5, 163.1, 163.0 (2C), 156.2, 152.6, 131.4, 129.7, 128.4, 127.9, 124.6, 123.6, 120.2, 119.5, 99.8, 79.1, 70.7, 70.6, 70.5, 70.4, 69.7, 68.5, 55.1 and 54.9 (8C), 49.9 (2C), 46.6 (2C), 40.8, 38.8 (2C), 38.6, 29.80 (2C), 28.60 (3C), 25.7 (2C); HR-MS (ESI-positive ion) *m/z* for $[C_{45}H_{70}N_8O_9]^{2+}$ $[M+2H]^{2+}$ calcd: 433.2628, found: 433.2624; IR ν_{max} (cm^{-1}) (oil) 3251 (w), 2872 (m), 1706 (m), 1636 (s).

Compound 7.

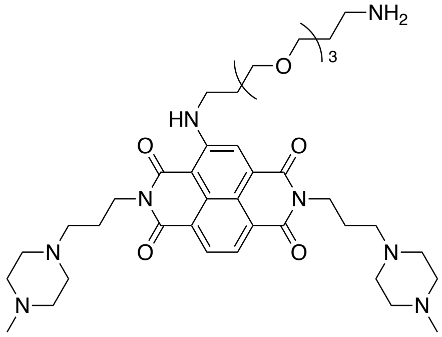

Boc-protected NDI **5** (230 mg, 0.26 mmol) was stirred in a solution of TFA in DCM (20%, 2.5 mL). The solution was stirred for 1 h at rt. Solvent was then removed under reduced pressure, and the crude solid then underwent multiple cycles of re-dissolution in MeOH followed by concentration under reduced pressure, to yield the Boc-deprotected product **7** (430 mg, quant.) as an octa-TFA salt. ^{1}H NMR (500 MHz, D_2O) δ 8.24 (d, J = 8.0, 1H), 8.00 (dd, J = 8.0, J = 1.5, 1H), 7.80 (s, 1H), 4.16 (dt, J = 13.0, J = 7.0, 4H), 3.79 – 3.63 (m, 12H), 3.65 – 3.41 (br. m, 18H), 3.30 – 3.23 (m, 4H), 3.12 (t, J = 7.0, 2H), 2.98 (app. d, J = 3.5, 6H), 2.18 – 2.06 (m, 6H) 1.97 (p, J = 6.5, 2H); ^{13}C NMR (126 MHz, D_2O) δ 165.2, 163.9, 163.6, 163.2, 152.0, 130.8, 128.5, 126.7, 125.1, 124.1, 122.0, 119.8, 118.4, 98.4, 69.6, 69.6, 69.4, 69.4, 68.2 (2C), 54.5, 54.4, 50.8 (2C), 50.7 (2C), 48.9 (2C), 48.9 (2C), 42.8 (2C), 40.1, 37.5 (3C), 28.5, 26.5, 22.6 (2C); HR-MS (MALDI) *m/z* for $[C_{40}H_{59}N_8NaO_7]^+$ $[M+Na]^+$ calcd: 787.4478, found: 787.4486; IR ν_{max} (cm^{-1}) 2882 (w), 2455 (w), 1778 (w), 1668 (w), 1639 (m), 1127 (s).

Compound 6

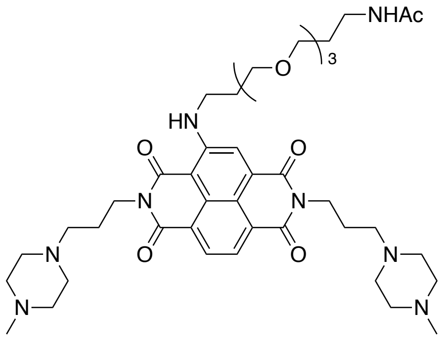

A solution of *N*-(3-(2-(2-(3-aminopropoxy)ethoxy)ethoxy)propyl)acetamide (**4**)[2] (di-TFA salt, 23.5 mg, 48 μmol) in Et_3N (0.5 mL) was added to a suspension of bromo-NDI **2** (8 mg, 13 μmol) in ACN (1 mL) under a nitrogen atmosphere. The reaction mixture was heated to 70 °C and stirred for 4 h. The solution was then cooled to rt, and solvent was removed under reduced pressure. The crude product was purified by HPLC (5% to 100% ACN w/0.05% TFA in H_2O w/0.05% TFA). ACN and TFA were removed under reduced pressure, which was followed by lyophilisation to yield NDI derivative **6** (11 mg, quant.) as a red solid. ^{1}H NMR (400 MHz, MeOD) δ 8.33 (d, J = 8.0, 1H), 8.06 (d, J = 8.0, 1H), 8.00 (s, 1H), 4.22 – 4.16 (m, 4H), 3.78 – 3.66 (m, 10H), 3.62 – 3.57 (m, 2H), 3.53 – 3.33 (m, 18H), 3.21 (t, J = 7.0, 2H), 3.14 – 3.06 (m, 4H), 2.91 (app. d, J = 1.5, 6H), 2.19 – 2.05 (m, 6H), 1.90 (s, 3H), 1.77 – 1.68 (m, 2H); ^{13}C NMR (101 MHz, MeOD) δ 173.1, 166.9, 164.6, 164.2, 164.1, 153.4, 130.4, 128.9, 127.1, 125.0, 124.1, 122.5, 120.7, 120.2, 100.1, 71.6 (2C), 71.5, 71.3, 69.9, 69.6, 55.9 (2C), 53.0 (2C), 52.9 (2C), 50.5 (4C), 43.6 (2C), 41.7, 39.3 (2C), 38.0, 30.5, 30.4, 24.9 (2C), 22.6; HR-MS (ESI-positive ion) *m/z* for $[C_{42}H_{63}N_8O_8]^+$ $[M+H]^+$ calcd: 807.4764, found: 807.4754; IR ν_{max} (cm^{-1}) 3280 (w), 2873 (w), 2416 (w), 1668 (s), 1639 (s).

NDI-CDs 9

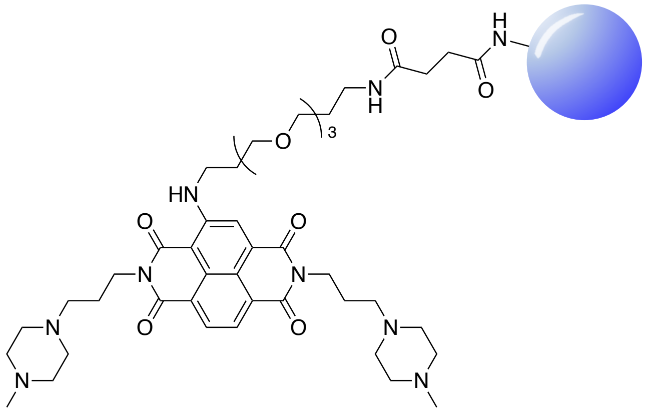

HATU (8 mg) and DIPEA (5 µL) were added to a solution of NDI **7** (13 mg) in DMF (1 mL) at rt under N_2. CDs **8** (10 mg) were added, and the solution was stirred for 24 h. Solvent was removed under reduced pressure, and the crude residue was redissolved in an aqueous solution of NaOH (0.1 M, 2 mL) and stirred for 1 h. The solution was then diluted with H_2O (8.5 mL) and washed with Et_2O. The aqueous extracts were then neutralised with HCl (1 M) and washed with Et_2O. H_2O was then removed from the aqueous extracts under reduced pressure, then the crude residue was redissolved in H_2O and dialysed for 48 h, prior to lyophilisation to yield the NDI-CDs **9** conjugate (14 mg).

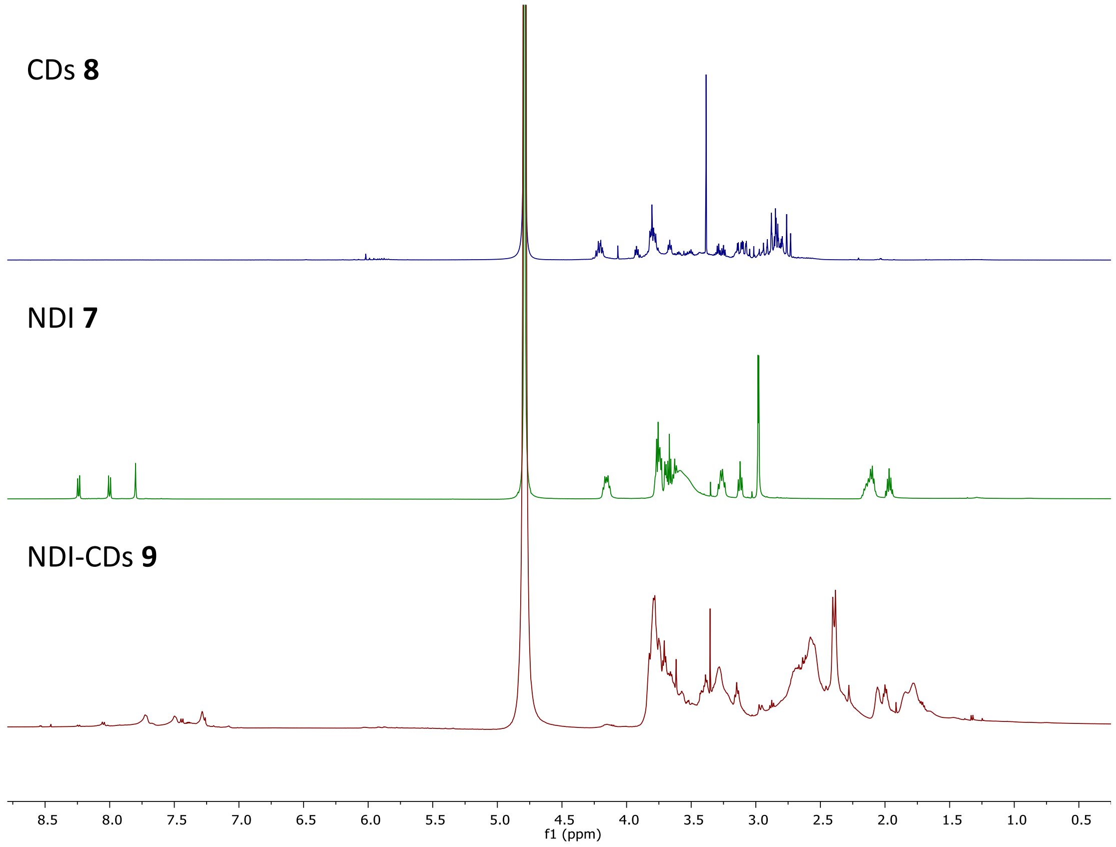

Figure S1. Superposition of ^{1}H NMR spectra of **8**, **7** and **9**.

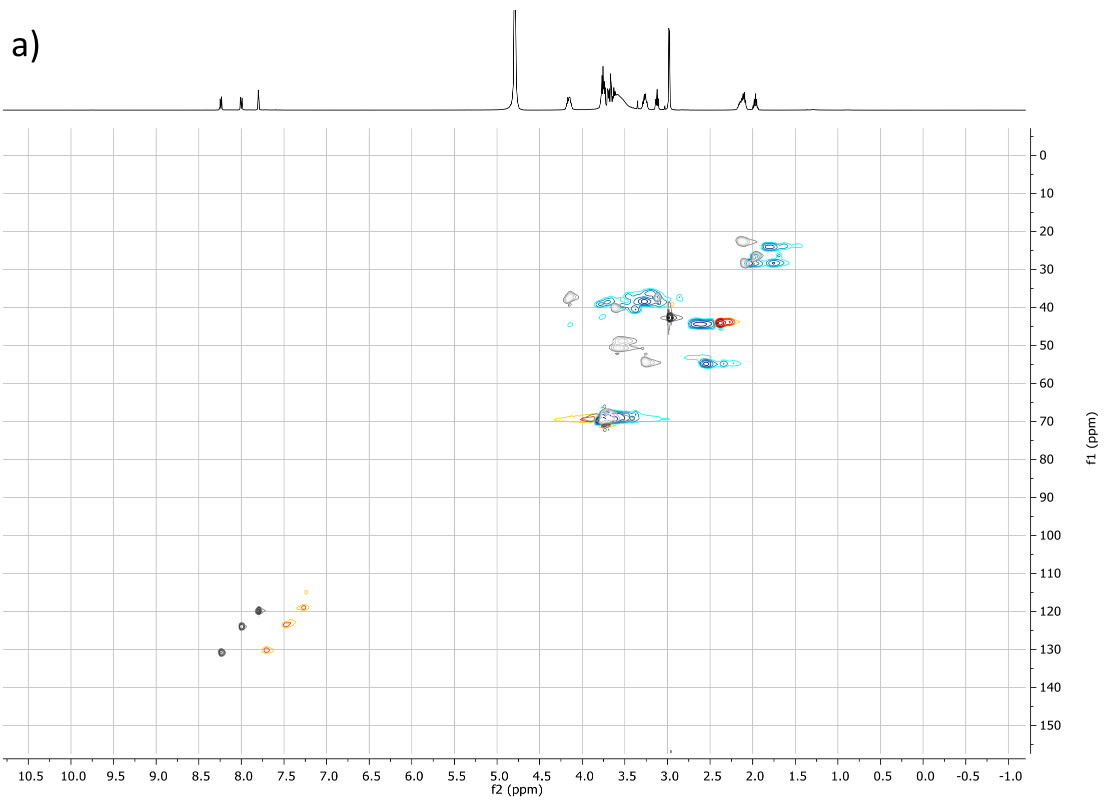

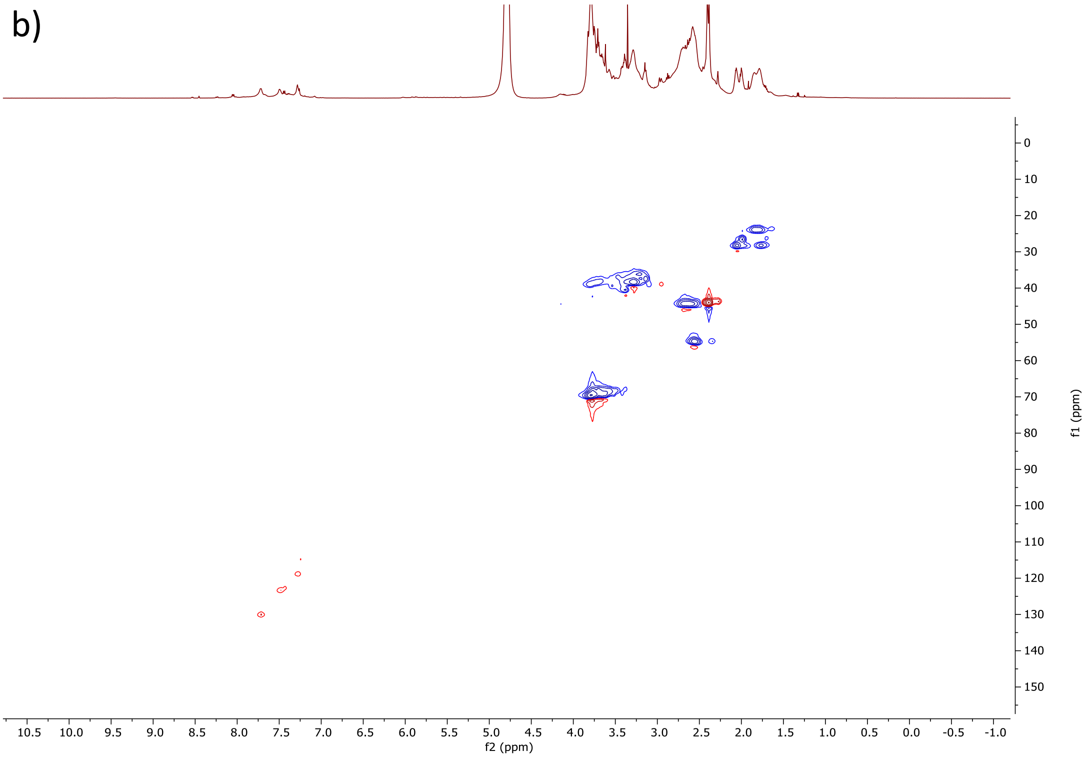

Figure S2. a) Overlaid HSQC (D_2O) spectra of **7** (greyscale) and **9** (orange-cyan gradient), with horizontal 1D ^1H NMR trace of **7** shown. b) HSQC (D_2O) spectrum of **9**

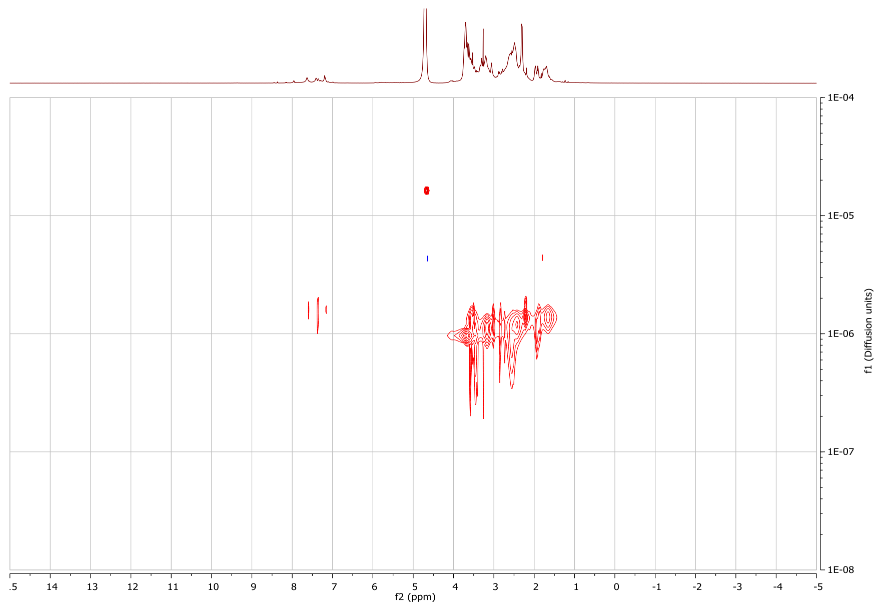

Figure S3. DOSY NMR spectrum of NDI-CDs **9** in D_2O used to calculate hydrodynamic radius. Chemical shift (ppm) versus diffusion rate (cm^2/sec).

Table S1. Diffusion coefficients and corresponding average hydrodynamic radii determined for CDs **8** and NDI-CDs **9**. Three diffusion coefficients were taken for each compound (a max, mid and min), and the hydrodynamic radius was calculated for each of these. The average radius is given, with the standard error of mean given.

	**Diffusion coefficient (x 10^{-6} cm^2/sec)**	**Hydrodynamic radius (nm)**
**8**	3.7, 2.5, 2.1	0.9 ± 0.1
**9**	2.0, 1.3, 0.9	1.8 ± 0.4

Quantitative NMR experiment. Maleic acid was used as an internal calibrant, and the wt.% of NDI **7** present in NDI-CDs **9** was calculated using Eq. 1,[3] in which; "t" is the target molecule (**7**) within the sample (**9**), and the internal calibrant (IC) is maleic acid; "P" is equal to purity in percent; "Int" is equal to NMR integral; "n" is equal to the number of nuclei of that integral; "MW" is equal to molecular weight in grams per mole; and "m" is equal to the mass in grams. This equation is used to determine the mass of a compound of known structure and molecular weight, within an accurately weighed sample, it is ideal for determining the mass of **7** within **9** because it does not require knowledge of the molecular weight of 'impurities' which in this case would be the CDs.[3] It was found that **9** is composed of 55 wt.% of **7**.

$$P\,[\%] = \frac{n_{IC} * Int_t * MW_t * m_{IC}}{n_t * Int_{IC} * MW_{IC} * m_s} * P_{IC} \qquad \text{Eq. 1}$$

*Synthesis of NDI-CDs **10***

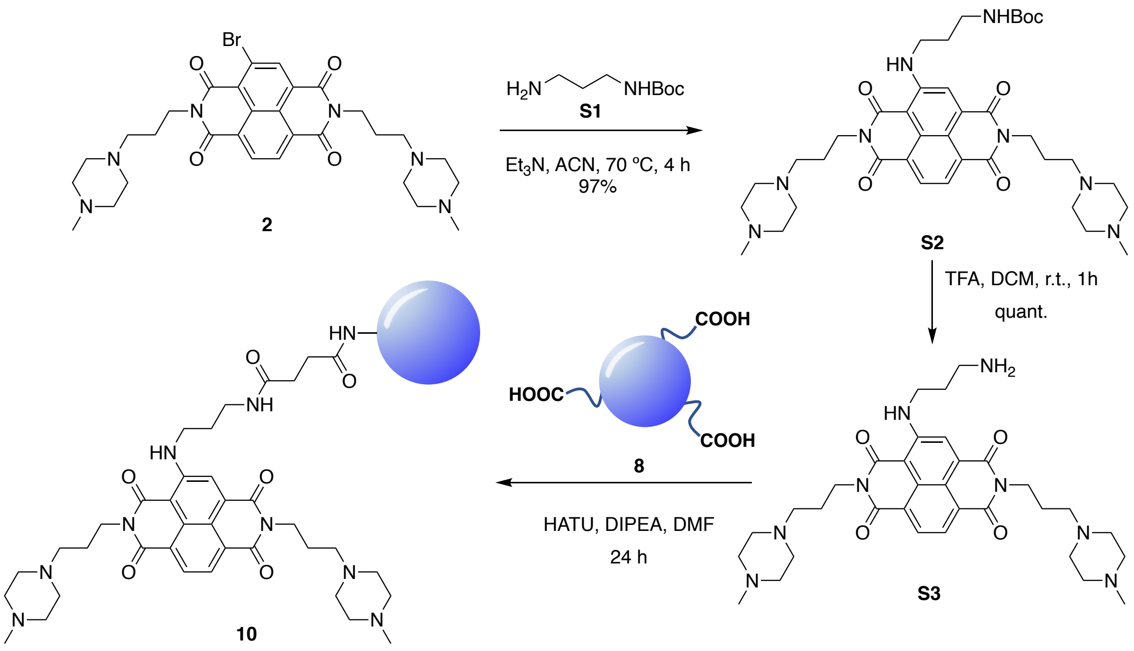

Compound S2

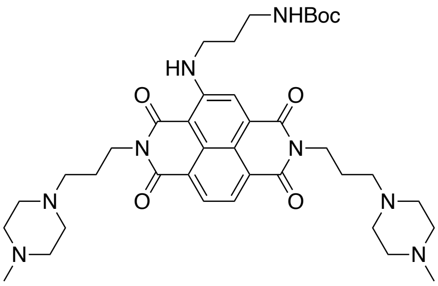

A solution of *tert*-butyl (3-aminopropyl)carbamate (**S1**)[4] (240 mg, 1.63 mmol) in Et_3N (2.7 mL) was added to a suspension of bromo-NDI **2** (340 g, 0.54 mmol) in ACN (5.5 mL) under a nitrogen atmosphere. The reaction mixture was heated to 70 °C and stirred for 4 h. The solution was then cooled to rt, and solvent was removed under reduced pressure. The crude product was redissolved in DCM and washed with H_2O. The organic extracts were dried with $MgSO_4$, and the solvent was removed under reduced pressure. The crude product was purified by flash column chromatography on silica (20% MeOH in DCM) to yield trisubstituted NDI **S2** (380 mg, 97%) as a solid. ^{1}H NMR (400 MHz, $CDCl_3$) δ 10.13 – 10.02 (m, 1H), 8.62 – 8.54 (m, 1H), 8.32 – 8.24 (m, 1H), 8.13 (s, 1H), 4.86 (br. s, 1H), 4.21 (t, J = 7.5, 4H), 3.62 (app. q, J = 6.5, 2H), 3.34 (app. q, J = 6.5, 2H), 2.49 (t, J = 7.0, 4H), 2.41 – 2.26 (br. m, 16H), 2.22 (s, 3H), 2.19 (s, 3H), 2.07 – 1.98 (m, 2H), 1.91 (p, J = 7.0, J = 6.5, 4H), 1.45 (s, 9H); ^{13}C NMR (101 MHz, $CDCl_3$) δ 166.2, 163.4, 163.0 (2C), 156.2, 152.3, 131.4, 129.5, 128.0, 126.3, 124.6, 123.6, 119.6, 119.4, 100.1,

79.7, 56.1, 56.0, 55.2 and 53.1 (8C), 46.1 (2C), 40.8, 39.5, 38.9, 38.2, 30.1, 28.5 (3C), 25.2, 25.0; HR-MS (ESI-positive ion) *m/z* for $[C_{38}H_{55}N_8O_6]^+$ $[M+H]^+$ calcd: 719.4240, found: 719.4217; IR ν_{max} (cm^{-1}) (compressed solid) 3364 (w, br.), 2934 (w), 1665 (s), 1635 (s).

Compound S3

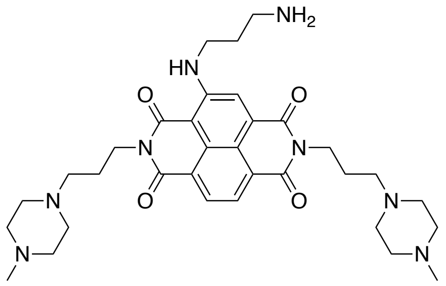

Boc-protected NDI **S2** (200 mg, 0.28 mmol) was stirred in a solution of TFA in DCM (20%, 2.5 mL). The solution was stirred for 1 h at rt. Solvent was then removed under reduced pressure, and the crude solid then underwent multiple cycles of re-dissolution in MeOH followed by concentration under reduced pressure, to yield the Boc-deprotected product **S3** (430 mg, quant.) as an octa-TFA salt. ^1H NMR (500 MHz, D_2O) δ 8.36 (d, J = 8.0, 1H), 8.12 (d, J = 8.0, 1H), 7.93 (s, 1H), 4.21 (q, J = 7.0, 4H), 3.72 (t, J = 7.0, 2H) 3.79 – 3.50 (br. s, 16H), 3.47 – 3.37 (m, 4H), 3.25 – 3.20 (m, 2H), 3.03 (app. d, J = 2.5, 6H), 2.25 – 2.12 (m, 6H); ^{13}C NMR (126 MHz, D_2O) δ 165.5, 164.2, 163.9, 162.5, 152.0, 131.0, 128.7, 127.1, 126.2, 125.3, 124.4, 122.3, 119.9, 118.7, 99.1, 54.5 (2C), 50.2 and 48.8 (8C), 42.1 (2C), 39.8, 37.0 (3C), 26.7, 22.3 (2C); HR-MS (ESI-positive ion) *m/z* for $[C_{33}H_{44}N_4O_4]^+$ $[M+H]^+$ calcd: 619.3715, found: 619.3698; IR ν_{max} (cm^{-1}) (compressed solid) 3013 (w, br.), 1638 (s), 1669 (s).

NDI-CDs 10

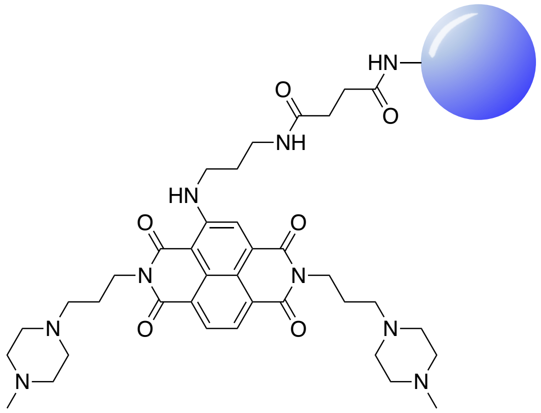

HATU (8.4 mg) and DIPEA (5 μL) were added to a solution of NDI **S3** (11 mg) in DMF (1 mL) at rt under N_2. CDs **8** (8.5 mg) were added, and the solution was stirred for 24 h. Solvent was

removed under reduced pressure, and the crude residue was redissolved in an aqueous solution of NaOH (0.1 M, 2 mL) and stirred for 1 h. The solution was then diluted with H_2O (8.5 mL) and washed with Et_2O. The aqueous extracts were then neutralised with HCl (1 M) and washed with Et_2O. H_2O was then removed from the aqueous extracts under reduced pressure, then the crude residue was redissolved in H_2O and dialysed for 48 hours, prior to lyophilisation to yield the NDI-CDs **10** conjugate (11 mg).

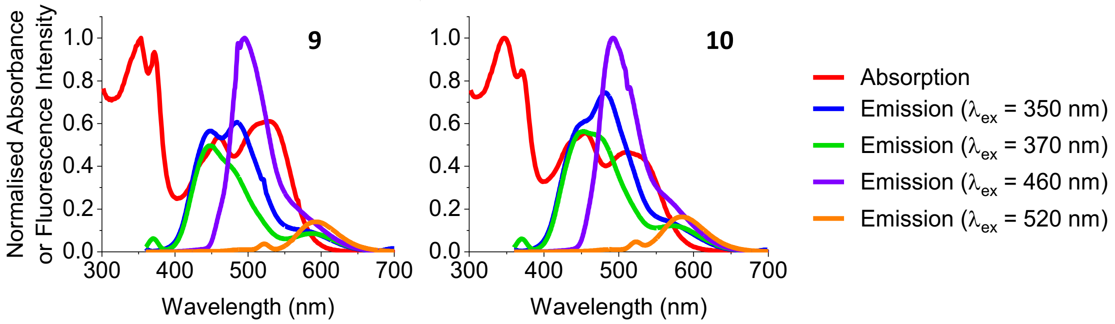

Figure S4. Overlaid absorption and emission spectra of NDI-CDs **9** and **10**.

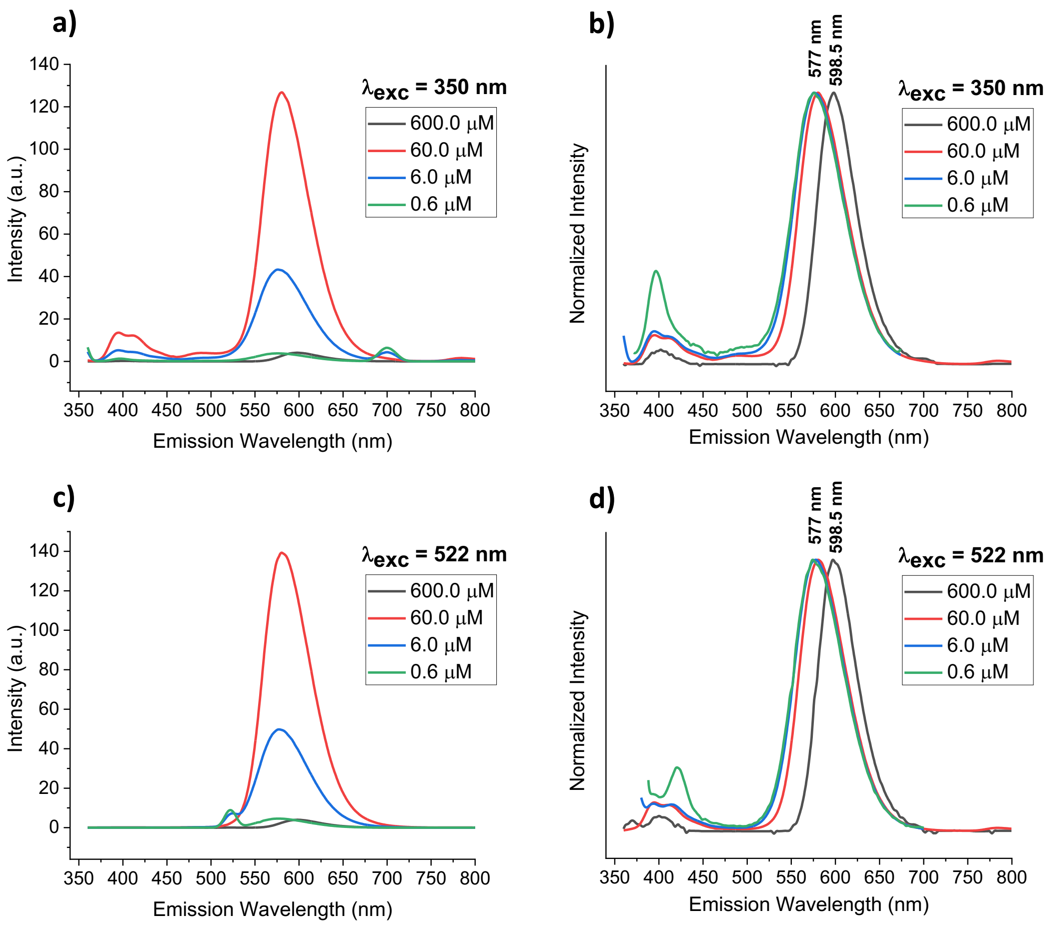

Figure S5. Fluorescence emission spectra of **7** at varying concentrations in water to show how λ_{max} changes with concentration. (a) λ_{ex} = 350 nm. (b) Normalised spectra, λ_{ex} = 350 nm. (c) λ_{ex} = 522 nm. (d) Normalised spectra, λ_{ex} = 522 nm.

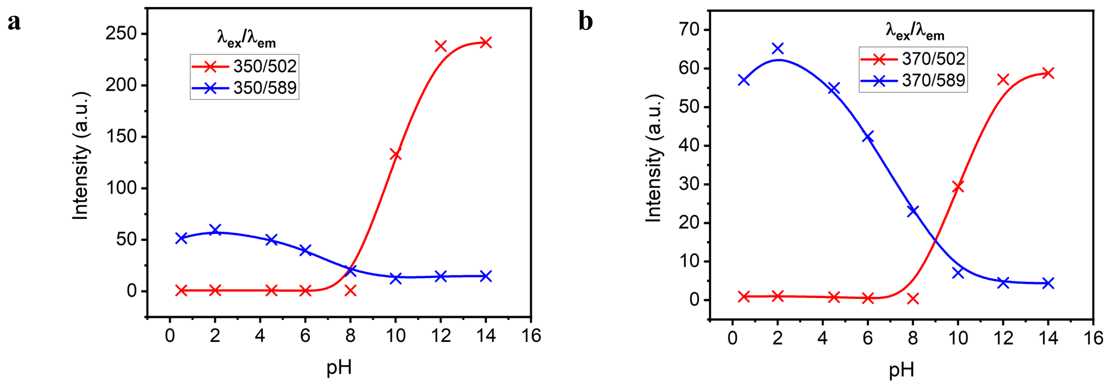

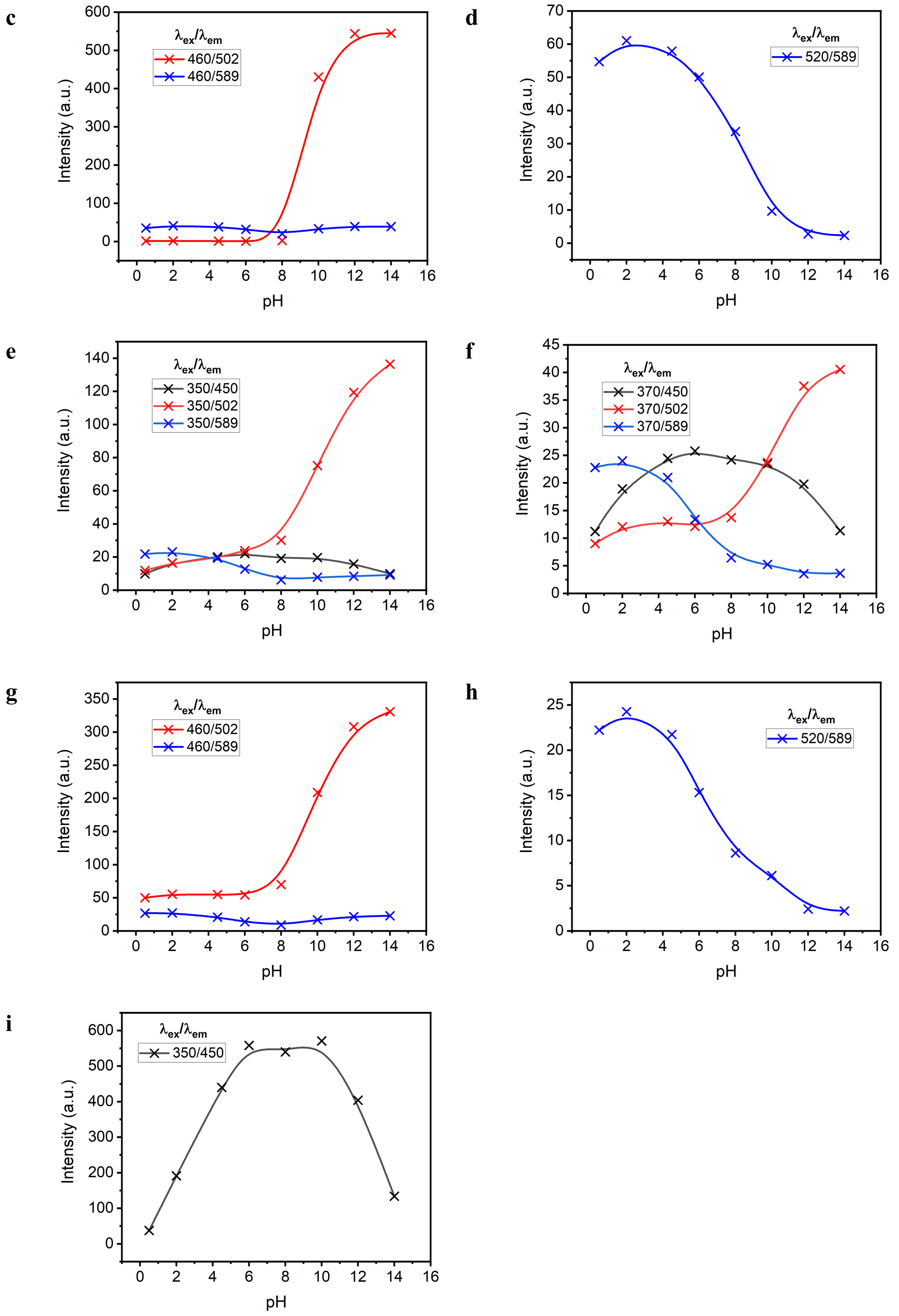

Figure S6. Change in fluorescence emission intensity of (a)-(d) **7**, (e)-(h) **9** and of (i) **8** at specified excitation and emission wavelengths and pHs.

3. Biophysical Studies

Oligonucleotides telo23 (human telomeric G-quadruplex): 5'-GGGTTAGGGTTAGGGTTAGGG-3' and ds26 (duplex DNA): 5'-TATAGCTATA-HEG-TATAGCTATA-3' (HEG = [(-CH_2CH_2O-)$_6$]) were purchased from Eurogentec (Belgium), purified by HPLC, and delivered dry. Oligonucleotide solutions were prepared by diluting with the appropriate amount of water or buffer according to the manufacturer's instructions. All oligonucleotides were annealed prior to use by heating to 90 °C for 2 min then immediately cooling in ice for 10 min.

3.1 Circular Dichroism titrations

Circular dichroism spectroscopy titration measurements were collected on a Jasco J-810 spectrometer fitted with a Peltier temperature controller which was set to 20 °C. A quartz cuvette with a path length of 5 mm was used for the titrations. Circular dichroism spectroscopy measurements were recorded between 320 – 220 nm, at a scan speed of 1000 nm/min, at intervals of 1 nm and a bandwidth of 1 nm.

Oligonucleotide sequences (telo23 or ds26) were used at a concentration of 5 µM in 1 mL of 100 mM potassium phosphate buffer pH 7.4. Ligand **6** stock solutions were 1 mM in 100 mM potassium phosphate buffer pH 7.4, and NDI-CDs **9** were 1 mg/mL in 100 mM potassium phosphate buffer pH 7.4, equivalent to 0.7 mM. Aliquots of the appropriate ligand or NDI-CDs stock solution were added to the appropriate oligonucleotide solution, with the circular dichroism spectrum measured after each addition. The reported spectrum for each sample is an average of three scans. Data processing was carried out using Origin® 2019b software. A smoothing polynomial was applied to all spectra. Observed ellipticities in mdeg were converted to molar ellipticity [θ] = deg cm^2 $dmol^{-1}$. Each titration was repeated in duplicate or triplicate. Control experiments were carried out in the absence of DNA for all ligands.

3.2 UV/Visible titrations

UV/visible spectra were recorded on an Agilent Cary 60 UV-Vis Spectrophotometer at rt. A 3 mL quartz cuvette with a path length of 10 mm was used for the titrations. UV/visible spectra were recorded between 700 – 300 nm, and baseline corrected for the buffer used. The ligand solution was kept constant throughout the titration (10 µM) in 100 mM potassium phosphate buffer pH 7.4 at an initial volume of 1.5 mL. Aliquots of oligonucleotide (telo23 or ds26) were added from a stock solution (100 µM oligonucleotide, 10 µM ligand to maintain constant ligand

concentration, 100 mM potassium phosphate pH 7.4). The UV/visible spectrum was measured after each addition. Each titration was repeated in duplicate or triplicate.

3.3 Fluorescence titrations

Fluorescence measurements were recorded on a PerkinElmer LS-45 Fluorimeter at rt. A 3 mL quartz cuvette with a path length of 10 mm was used for the titrations. Samples were excited at 370 nm, and the emission spectrum was measured from 400-700 nm. The ligand solution was kept constant throughout the titration (**6** = 10 µM, **9** = 10 µg/mL or 7.2 µM) in 100 mM potassium phosphate buffer pH 7.4 at an initial volume of 1.5 mL. Aliquots of oligonucleotide (telo23 or ds26) were added from a stock solution (100 µM oligonucleotide, 10 µM **6**/ 10 µg/mL or 7.2 µM **9** to maintain constant ligand concentration, 100 mM potassium phosphate pH 7.4). The emission spectrum was measured after each addition. Each titration was repeated in duplicate or triplicate. Data was processed using the non-linear curve fitting tool in Origin® 2019b software following a binding model to Eq. 2, in the case of a titration of ligand (L) with DNA (D), resulting in complex formation (D·L). The degree of complex formation at equilibrium is quantified by association constant ($K_a = 1/K_d$, where K_d is dissociation constant), which can be calculated from the change in the measured fluorescence intensity (Δ Intensity).

$$\Delta\, Intensity = \varepsilon_{\Delta D.L} \cdot \frac{\left([D]_{tot} + [L]_{tot} + \frac{1}{K_a}\right) - \sqrt{\left([D]_{tot} + [L]_{tot} + \frac{1}{K_a}\right)^2 - 4 \cdot [D]_{tot}\,[L]_{tot}}}{2} \qquad \text{Eq. 2}$$

3.4 FRET Melting Assays

Fluorescence resonance energy transfer (FRET) melting assays were performed according to the procedure reported by De Cian and co-workers[5] on Roche LightCycler 480 qPCR instrument. In these assays, oligonucleotides of interest were obtained labelled at the 5' and 3' ends with FAM (a fluorescence donor) and TAMRA (a fluorescence quencher), respectively. In the folded state, proximity of the donor and quencher result in no observed fluorescence from FAM, since energy is transferred non-radiatively to TAMRA by FRET. As the temperature is raised and the secondary structure denatures, the fluorophores move further apart and hence the fluorescence signal increases. From the resulting curve, the characteristic melting temperature ($T_{1/2}$) is defined as that at which the normalised fluorescence signal equals 0.5. The change in melting temperature (ΔT_m) induced by a small molecule ligand compared to that of the oligonucleotide in the absence of ligand provides an indication of the ligand's ability to stabilise the G4 structure.

The method consisted of holding at 25 °C for 5 min, before heating at 1 °C/min to 96 °C in 1 °C increments, followed by monitoring the fluorescence output at each increment for 1 min. The fluorescence emission of FAM was followed at 516 nm, with a 10 nm full width at half-maximum filter and an 8-fold gain, after excitation at 492 nm with a 9 nm full width at half-maximum filter. All oligonucleotides used were purchased from Eurogentec (Belgium), purified by HPLC and delivered dry (Table S2). Oligonucleotide concentrations were determined by UV-absorbance using a NanoDrop 2000 Spectrophotometer from Thermo Scientific. All sequences were annealed before use by heating for 2 min at 90°C and then placed immediately into ice. The final concentration of oligonucleotide was 200 nM in all cases. The buffer used depended on the sequence in question: 10 mM KCl, 90 mM LiCl and 10 mM Li cacodylate for Febr1T-K^+, FhtelT-K^+ and F10T-K^+; 100 mM NaCl and 10 mM Li cacodylate for FhtelT-Na^+; and 1 mM KCl, 99 mM LiCl and 10 mM Li cacodylate for FmycT-K^+. Ligand concentrations were either 1, 2, 5 or 10 µg/mL. Each sample was tested in duplicate on the same plate, and each plate was repeated in at least duplicate to assess the reproducibility of all results.

Appropriate control experiments were also carried out for each sample set. Data processing was carried out using Origin 9, with $\Delta T_{1/2}$ used to represent ΔT_m.

Table S2. Oligonucleotides employed in FRET melting assays

DNA model	Sequence
**Febr1T (T.brucei G4)**	5'-FAM-GGGCAGGGGGTGATGGGGAGGAGCCAGGG-TAMRA-3'
**Ftel22T (human telomeric G4)**	5'-FAM-GGGTTAGGGTTAGGGTTAGGG-TAMRA-3'
**FmycT (c-myc promoter G4)**	5'-FAM-TTGAGGGTGGGTAGGGTGGGTAA-TAMRA-3'
**F10T (duplex)**	5'-FAM-TATAGCTATA-HEG-TATAGCTATA-TAMRA-3'

FAM = 6-carboxyfluorescein; TAMRA = 6-carboxy-tetramethylrhodamine; HEG = [(-CH_2CH_2O-)$_6$

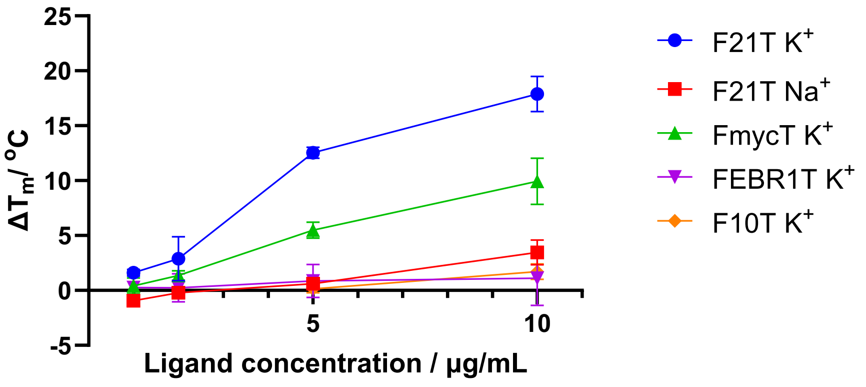

Figure S7. DNA stabilisation of **9** assessed via FRET Melting Assay at 1, 2, 5 and 10 µg/mL ligand and 200 nM DNA concentration.

4. Biological studies

4.1 Cell culture

Cell culture media, additives and other relevant consumables were purchased from Invitrogen, Life Technologies. Cell culture was carried out in a laminar flow hood following standard aseptic techniques. HeLa (human cervical cancer cell line) were grown in Dulbecco's Modified Eagle Medium (DMEM) with high glucose (4.5 g/L). HDF (human dermal fibroblast, healthy cell line) were grown in Minimum Essential Medium (MEM) with Earle's Salts. All growth media were supplemented with GlutaMax™, 10% fetal bovine serum (FBS), and an antibiotic-antimycotic cocktail (AntiAnti). Confluent cultures were detached from the surface using trypsin (Tryp LE Express) and cells were plated in individual glass-bottomed wells (for confocal imaging) or 96-well plates (for toxicity analysis). Cells were kept in an incubator at 37 °C in an atmosphere of 5% CO_2. After plating, cells were left for 24 h to allow for adhesion to the surface before any experiments were carried out.

4.2 Cytotoxicity

Cells plated in 96-well plates in a volume of 100 µL with 2000 HeLa cells/10000 HDF cells per well. Cytotoxicity was determined by assay with AlamarBlue. After desired incubation time with the compound (0.1-320 µg/mL), the media was removed, wells washed with PBS twice and a solution of AlamarBlue (5%) in serum-free media was added to each well. The plates were then incubated for 2 h at 37 °C in an atmosphere of 5% CO_2 before the fluorescence was measured on a BMG Labtech CLARIOstar plate reader (Excitation – 370 nm, Emission – 590 nm). The cell viability was then determined each concentration as a percentage of untreated cells, and the average viability was calculated from 6 repeats. The non-linear dose-response fitting tool in Prism was then used to determine IC_{50} values.

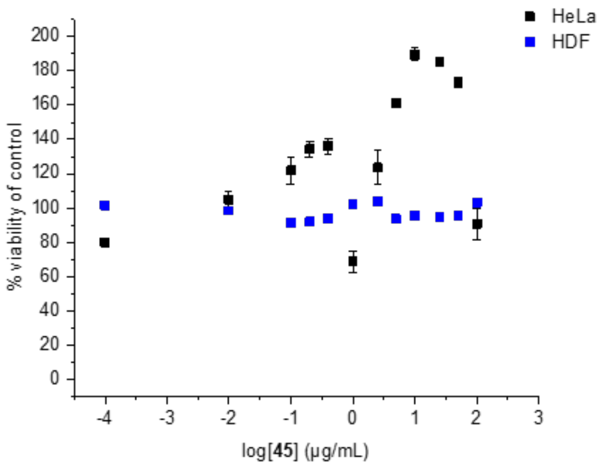

Figure S8. Effects on the number of live cells (Alamar Blue assay) for HeLa and HDF cell lines after 72 h incubation with CDs **8.**

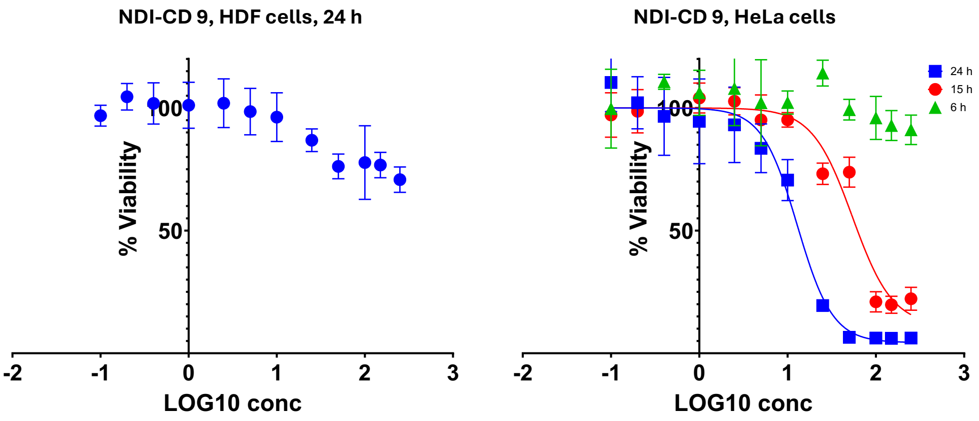

Figure S9. Alamar Blue assay after 6-24 h. Significant toxicity observed in HeLa within minimal in HDF cells for NDI-CD **9** treated HDF and HeLa cells at 6 h. Concentrations in µg/mL.

4.3 Confocal microscopy

Hela cells were plated in a 24-well plate (100 K cells per well) and incubated with NDI-CDs **9** (250 µg/mL) for 2 h. After incubation, the cells were washed thrice with PBS and then stained with one of three stains as per manufacturer's protocols: nuclear ID DNA stain (1x, 15 min), mitotracker Deep Red FM (770 nM, 30 min), Lysotracker blue DND22 (100 nM, 30 min). The cells were then fixed in formaldehyde (4% in PBS, 10 min, 0 °C), washed twice with PBS then stored in PBS at 0 °C until imaging. Mounting and confocal imaging was carried out by Dr Katy Jepson in the Wolfson Bioimaging Facility on a Leica SP8 AOBS confocal laser scanning microscope attached to a Leica DMi8 inverted epifluorescence microscope using a HC PL APO

CS2 63x/1.40 OIL lens at 2x zoom. λ_{ex} 460 nm/ λ_{em} 500 nm. Images were processed using Fiji (ImageJ). Pearson's correlation values were determined with the Coloc-2 tool in Fiji.

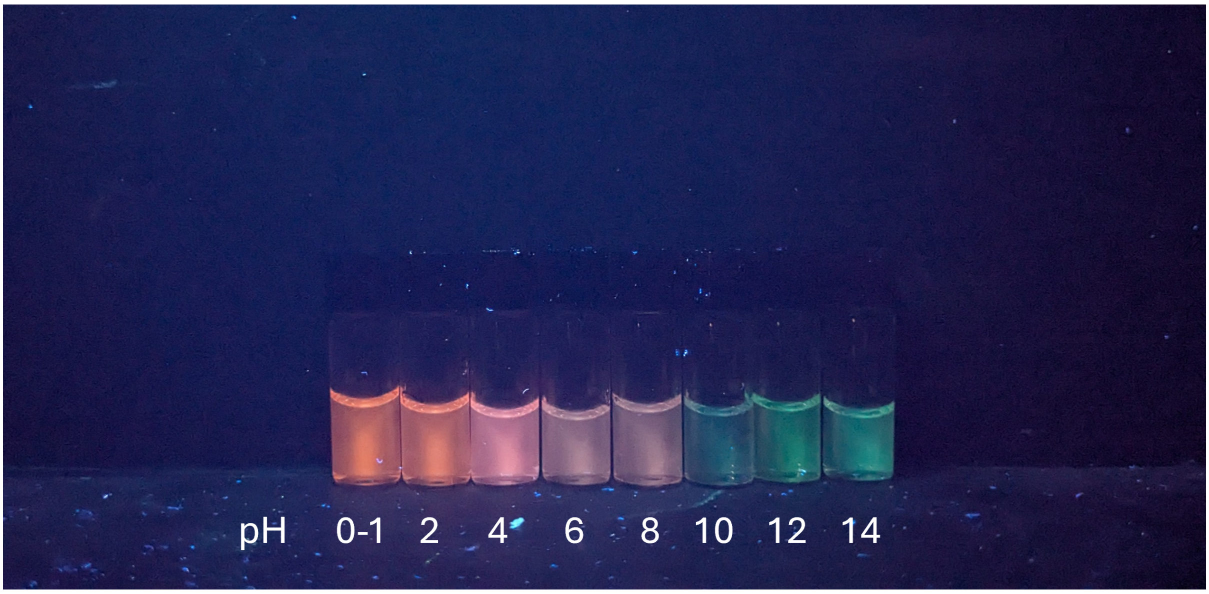

Figure S10. Image of NDI-CDs **9** in aqueous solutions at different pHs (λ_{em}=365 nm).

5. References

1. X. Hou, Q. Yu, F. Zeng, C. Yu and S. Wu, *Chem Commun (Camb)*, 2014, **50**, 3417-3420.
2. D. Pulido, V. Casado-Anguera, L. Perez-Benito, E. Moreno, A. Cordomi, L. Lopez, A. Cortes, S. Ferre, L. Pardo, V. Casado and M. Royo, *J Med Chem*, 2018, **61**, 9335-9346.
3. G. F. Pauli, S.-N. Chen, C. Simmler, D. C. Lankin, T. Gödecke, B. U. Jaki, J. B. Friesen, J. B. McAlpine and J. G. Napolitano, *Journal of Medicinal Chemistry*, 2014, **57**, 9220-9231.
4. D. Muller, I. Zeltser, G. Bitan and C. Gilon, *The Journal of Organic Chemistry*, 1997, **62**, 411-416.
5. A. De Cian, L. Guittat, M. Kaiser, B. Sacca, S. Amrane, A. Bourdoncle, P. Alberti, M. P. Teulade-Fichou, L. Lacroix and J. L. Mergny, *Methods*, 2007, **42**, 183-195.
